# Supplementary material for: Early-onset burdensome multimorbidity: an exploratory analysis of sentinel conditions, condition accrual sequence and duration of three long-term conditions using the 1970 British Cohort Study
Source: BMJ Open. 2022 Oct 10;12(10):e059587. doi: 10.1136/bmjopen-2021-059587 (PMC9557794; doi:10.1136/bmjopen-2021-059587)
Supplement: Supplementary data [file bmjopen-2021-059587supp001.pdf]

Supplementary Materials

Table 1. Ethnicity of the cohort members at age five[1] for MLTC-M group (psychological distress, high blood pressure and back pain), compared with remainder of BCS70 sample at age 46.

		MLTC-M	Non MLTC-M
		N (%)	N (%)
Ethnicity	White British	276 (94.5%)	6268 (96.7%)
	European	5 (1.7%)	58 (0.9%)
	West Indian	4 (1.4%)	52 (0.8%)
	Indian-Pakistani	5 (1.7%)	77 (1.2%)
	Other Asian	0 (0%)	8 (0.1%)
	African	1 (0.3%)	1 (0.0%)
	Other	1 (0.3%)	16 (0.25%)
	Total	292 (100%)	6480 (100%)

[1]Ethnicity of the cohort member was not recorded at birth, age 5 was the first sweep where ethnicity was reported.
