# Supplementary material for: Early-onset burdensome multimorbidity: an exploratory analysis of sentinel conditions, condition accrual sequence and duration of three long-term conditions using the 1970 British Cohort Study
Source: BMJ Open. 2022 Oct 10;12(10):e059587. doi: 10.1136/bmjopen-2021-059587 (PMC9557794; doi:10.1136/bmjopen-2021-059587)
Supplement: Author's manuscript [file bmjopen-2021-059587.draft_revisions.pdf]

BMJ Open

BMJ Open is committed to open peer review. As part of this commitment we make the peer review history of every article we publish publicly available.

When an article is published we post the peer reviewers' comments and the authors' responses online. We also post the versions of the paper that were used during peer review. These are the versions that the peer review comments apply to.

The versions of the paper that follow are the versions that were submitted during the peer review process. They are not the versions of record or the final published versions. They should not be cited or distributed as the published version of this manuscript.

BMJ Open is an open access journal and the full, final, typeset and author-corrected version of record of the manuscript is available on our site with no access controls, subscription charges or pay-per-view fees (http://bmjopen.bmj.com).

If you have any questions on BMJ Open's open peer review process please email info.bmjopen@bmj.com

BMJ Open

Early-onset burdensome multimorbidity: an exploratory analysis of sentinel conditions, condition accrual sequence and duration using the 1970 British Cohort Study

Journal:	*BMJ Open*
Manuscript ID	bmjopen-2021-059587
Article Type:	Original research
Date Submitted by the Author:	24-Nov-2021

Complete List of Authors:	Stannard, Sebastian; University of Southampton, School of Primary Care, Population Sciences and Medical Education<br>Holland, Emilia; University of Southampton, School of Primary Care, Population Sciences and Medical Education<br>Crozier, S; University of Southampton Faculty of Medicine, 2. MRC Lifecourse Epidemiology Centre; NIHR, Applied Research Collaboration Wessex<br>Hoyle, Rebecca; University of Southampton, School of Mathematical Sciences<br>Boniface, Michael; University of Southampton, IT Innovation Centre<br>Ahmed, Mazen; University of Southampton, IT Innovation Centre<br>McMahon, James; NIHR, Applied Research Collaboration Wessex<br>Ware, William; NIHR, Applied Research Collaboration Wessex<br>Zlatev, Zlatko; University of Southampton, IT Innovation Centre<br>Alwan, Nisreen; University of Southampton, Academic unit of Primary Care and Population Sciences, Faculty of Medicine; University of Southampton, NIHR Southampton Biomedical Research Centre<br>Fraser, Simon; University of Southampton, School of Primary Care, Population Sciences and Medical Education

Keywords:	EPIDEMIOLOGY, Back pain < ORTHOPAEDIC & TRAUMA SURGERY, Hypertension < CARDIOLOGY, MENTAL HEALTH

SCHOLARONE™
Manuscripts

I, the Submitting Author has the right to grant and does grant on behalf of all authors of the Work (as defined in the below author licence), an exclusive licence and/or a non-exclusive licence for contributions from authors who are: i) UK Crown employees; ii) where BMJ has agreed a CC-BY licence shall apply, and/or iii) in accordance with the terms applicable for US Federal Government officers or employees acting as part of their official duties; on a worldwide, perpetual, irrevocable, royalty-free basis to BMJ Publishing Group Ltd ("BMJ") its licensees and where the relevant Journal is co-owned by BMJ to the co-owners of the Journal, to publish the Work in this journal and any other BMJ products and to exploit all rights, as set out in our licence.

The Submitting Author accepts and understands that any supply made under these terms is made by BMJ to the Submitting Author unless you are acting as an employee on behalf of your employer or a postgraduate student of an affiliated institution which is paying any applicable article publishing charge ("APC") for Open Access articles. Where the Submitting Author wishes to make the Work available on an Open Access basis (and intends to pay the relevant APC), the terms of reuse of such Open Access shall be governed by a Creative Commons licence – details of these licences and which Creative Commons licence will apply to this Work are set out in our licence referred to above.

Other than as permitted in any relevant BMJ Author's Self Archiving Policies, I confirm this Work has not been accepted for publication elsewhere, is not being considered for publication elsewhere and does not duplicate material already published. I confirm all authors consent to publication of this Work and authorise the granting of this licence.

For peer review only

Early-onset burdensome multimorbidity: an exploratory analysis of sentinel conditions, condition accrual sequence and duration using the 1970 British Cohort Study

Authors:

Sebastian Stannard[1], Emilia Holland[1], Sarah R Crozier[2,3], Rebecca Hoyle[4], Michael Boniface[5], Mazen Ahmed[5], James McMahon[7], William Ware[7], Zlatko Zlatev[5], Nisreen A Alwan[1,6,7] Simon DS Fraser[1]

1. School of Primary Care, Population Sciences and Medical Education, Faculty of Medicine, University of Southampton, UK
2. MRC Lifecourse Epidemiology Centre, Faculty of Medicine at the University of Southampton, UK
3. NIHR Applied Research Collaboration Wessex, Southampton Science Park, Innovation Centre, 2 Venture Road, Chilworth, Southampton, SO16 7NP
4. School of Mathematical Sciences, University of Southampton, Southampton, UK
5. University of Southampton, IT Innovation Centre, Southampton, UK
6. NIHR Southampton Biomedical Research Centre, University of Southampton and University Hospital Southampton NHS Foundation Trust, Southampton, UK
7. NIHR Applied Research Collaboration Wessex, Southampton, UK

Corresponding Author:

Sebastian Stannard

Murray Building (58)
59 Salisbury Road
Southampton
SO17 1TW

S.Stannard@soton.ac.uk

Word count: 4125

For peer review only

Abstract

Objectives: The prevalence of multiple long-term condition (LTC) multimorbidity is increasing with younger onset amongst socioeconomically deprived populations. Research on lifecourse trajectories towards multimorbidity is limited and early-onset multimorbidity poorly characterised. Understanding sentinel conditions (the first LTC occurring in the lifecourse), the sequence of LTC accrual and the permanency of the reporting of LTCs may help identify time points for prevention efforts. We utilised a longitudinal birth cohort to estimate the prevalence of the most common three-condition early-onset multimorbidity (MLTC-M) group at age 46, describe the frequency of sentinel conditions, the sequence of LTC accrual and explore the permanency of the reporting of LTCs.

Design: Retrospective cohort study using data from 6 adulthood 'sweeps' (rounds of data collection) at age 26, 30, 34, 38, 42 and 46.

Setting: 1970 British Cohort Study.

Participants: 17,196 cohort members born in 1970.

Outcome measures: Prevalence of the most common three-condition multimorbidity group at age 46. The nature and timing of sentinel conditions, the sequencing patterns of subsequent LTC accrual and the permanency of the reporting of psychological distress.

Results: At age 46 high blood pressure, psychological distress and back pain were the most common three-condition MLTC-M group, (n=370,4.3%). A subgroup of 164 (44.3%) people provided complete information on LTC. Psychological distress measured by the Malaise Index was the most common sentinel condition, occurring in 41 (25.0%), followed by back pain (n=36,22%). At age 26, 75/164 (45.1%) reported their sentinel condition. The most common sequence of LTC accrual was the co-reporting of psychological distress and back pain followed by high blood pressure. Almost a third (n=50,30.5%) reported a variation of psychological distress across the adult lifecourse.

Conclusion: In these exploratory analyses, psychological distress and back pain were the most common sentinel conditions. Birth cohorts may usefully inform life course-focused multimorbidity research.

Keywords: multimorbidity, long-term conditions, lifecourse, early-onset, birth cohort

Strengths and Limitations of this study:

- The use of a prospective longitudinal approach provides the opportunity to analyse the time-sequenced nature of the data.
- Knowing the temporal sequencing of long-term conditions enabled us to describe the frequency of sentinel conditions, the sequence of long-term condition accrual and explore the permanency of the reporting of a long-term condition.
- Using birth cohort data allowed us to explore and characterise sequences that incorporated the concept of 'burdensome' multimorbidity by combining both mental and physical conditions.
- The study was limited by sample size, wave-non response and no agreed definition of long-term condition.

For peer review only - http://bmjopen.bmj.com/site/about/guidelines.xhtml

- The self-reporting of long-term conditions and the lack of ethnic diversity resulted in the probable underreporting of multimorbidity.

For peer review only

For peer review only - http://bmjopen.bmj.com/site/about/guidelines.xhtml

INTRODUCTION

In the UK, the prevalence of multiple long-term condition multimorbidity (MLTC-M) has increased over the last two decades and is projected to continue to rise,[1]. This presents a significant challenge to health and prevention services,[2].

MLTC-M occurs earlier in the lifecourse among people from more socioeconomically disadvantaged backgrounds,[3]. While often considered a condition of older age, the majority of people living with multimorbidity are under 65,[3]. Earlier and greater accrual of long-term conditions (LTCs) may be influenced by the timing and nature of the exposure to key risk factors or other LTCs and the influence of wider determinants at different life stages.

Research on MLTC-M occurring by midlife remains limited,[4]. Growing evidence suggests that the sequence of accrual of LTCs varies considerably and influences outcomes,[5]. The sentinel condition (the first long-term condition occurring in the lifecourse in the development of MLTC-M), may play an important role. Sentinel conditions may significantly influence subsequent behaviour and other factors that influence the eventual cluster of LTCs, in turn influencing the nature and risk of important outcomes. Diagnosis of a condition is associated with actions such as medication prescription, specialist referral, and self-management advice, that may alter future trajectories of risk. For example, hypertension diagnosis leading to antihypertensive prescription and physical activity advice reducing future stroke risk. The time point of diagnosis therefore becomes an important determinant of future events,[6].

The sequence of LTC development may also be a key determinant of the nature of eventual LTC clusters,[7]. In a longitudinal analysis using UK primary care data from London, Ashworth et al identified that multimorbidity in those under 65 tended to start with depression or serious mental illness followed by combinations of obesity, chronic pain and diabetes,[7]. In contrast, for those aged 65 and over, diabetes and coronary heart disease were the predominant sentinel conditions, followed by combinations of chronic kidney disease, heart failure and stroke,[7]. Additionally, the prevalence of multimorbidity tends to rise steeply in midlife,[8] and middle-aged people with multimorbidity tend to have poorer health related quality of life,[9].

Across the life course, LTCs may not be permanent and may be of variable severity. For example, mental health conditions such as anxiety and depression are often not experienced as a 'fixed state',[10-12]. However, those with a persistent and chronic trajectory of depression tend to have poorer outcomes including suicide, a decrease in physical activity, ischemic heart disease and mortality,[13-16]. Using birth cohort data affords the opportunity to explore repeated validated measures of mental health across the lifecourse. Understanding the patterns of the reporting of mental health for those with MLTC-M may be important for identifying high-risk temporal life periods in the development of multimorbidity.

There also remains limited research on the differential burden experienced by patients with different multimorbidity clusters. Despite research suggesting the relationship between mental and physical health conditions is bidirectional,[17], there remains a need to understand what constitutes burden and complexity for people with multimorbidity. In particular, the National Institute for Health and Care Excellence multimorbidity guidelines recommend establishing better understanding of disease burden encompassing both complex disease and combined mental and physical health,[18].

For peer review only - http://bmjopen.bmj.com/site/about/guidelines.xhtml

Previous studies have used limited time frames and limited analyses of the sequence of accrual of LTCs,[4]. Using birth cohort data to study multimorbidity at midlife is under-explored. Gondek et al used data from the 1970 British Cohort Study (BCS70) suggesting that multimorbidity (two LTCs) is common at age 46-48 (34%),[19]. Building on this work, we aimed to study the most common three-condition multimorbidity group at age 46 using the BCS70 in order to explore and characterise sequences and incorporate the concept of 'burdensome' multimorbidity by combining both mental and physical conditions. We aimed to describe the nature, timing and recurrence of the sentinel condition for those reporting the most common three-condition MLTC-M, to illustrate the sequence of accrual of LTCs following the sentinel condition and describe the permanency of the reporting of psychological distress.

METHODS

Birth cohort study:

The BCS70 followed 17,096 participants born in 1970 from across England, Scotland and Wales. Since birth, data was collected in 'sweeps' roughly every four years resulting in 10 sweeps of data collection to date. Extensive information was collected on topics including socio-economic circumstances, family background, cognitive development, education, employment, partnerships, fertility, health and health-related behaviour,[20]. Data was collected using a range of survey methods including self-reported and measured outcomes. A user guide for the dataset can be found at the Centre for Longitudinal Studies,[21]. A cohort profile provides information about the background to the study and full survey methods for each sweep,[20,22].

Study sample:

Information was collected on 17,196 babies born in 1970. At age 46, 8581 (49.9%) of the original population at birth responded - 8536 provided information on LTCs. To derive the sentinel condition and accrual sequence we considered the MLTC-M group at age 46 who provided information across all the sweeps of data collection in adulthood (age 26, 30, 34, 38, 42 and 46). (n=164/370).

Patient and public involvement statement:

Patient and Public Involvement and Engagement (PPIE) co-investigators have been involved at every stage of the research process. Their lived experiences were important for informing the design of the research, incorporation the views of others and shaping our ideas. In particular PPIE input directed us to consider multimorbidity clusters that were both burdensome and complex and to consider the permanency of the reporting of LTCs.

Variables:

Sociodemographic measures at age 46:

A number of area-level and individual-level sociodemographic variables were recorded at age 46. Measures included sex (men/women), the Index of Multiple Deprivation (IMD) (in quintiles) and highest academic qualification (no qualification, GCSE or equivalent, A levels

For peer review only

or equivalent or degree and higher). Other measures included a three-measure index of housing tenure (own/mortgage, social rent and private rent/other), measure of local authority or housing association housing (yes/no), employment status (employed, unemployed, sickness/disability or caring for family) and a measure of receipt of benefits (yes/no).

MLTC-M outcome at age 46:

We selected psychological distress, back pain and high blood pressure as our MLTC-M group at age 46 because our scoping work suggested that this combination of three LTCs was the most common MLTC-M groups we considered (Table 1). The other nine three-condition MLTC-M groups encompassed the most common LTCs at age 46, including psychological distress, back pain, high blood pressure, asthma and arthritis. Psychological distress measured via the Malaise Index is not per se pathological and therefore not strictly morbidity but we use it an indicator of mental health. The most common three-condition MLTC-M group selected also explored the concept of 'burdensome' multimorbidity by combining both mental and physical conditions.

Long term conditions at ages 26, 30, 34, 38 and 42:

To explore the sentinel condition, sequence of accrual and the permanency of the reporting of psychological distress we required information on LTCs across all of the sweeps in adulthood (age 26, 30, 34, 38 and 42). All measures collected between ages 26 and 42 were self-reported and we had no measured blood pressure. If two or more LTCs were recorded at the same sweep we were unable to identify which condition occurred first.

Psychological distress at age 46 was classified if a cohort member had measured high on the Malaise Index,[23] or if they had visited a doctor or specialist for anxiety and/or depression in the last four years or since their last interview. The Malaise Index was selected because it was consistently present at sweeps in adulthood. The Malaise Index was self-reported and captures general psychological distress level, depression and anxiety symptoms,[24,25]. Scores above the cut off (4 or higher) represent a clinical diagnosis of common mental disorder,[24]. Scalar invariance of the measure was found within and across two birth cohort studies, as well as between genders,[26,27]. The Malaise Index has good psychometric properties,[28] and has been used both in the general population and high-risk groups,[29,30].

Psychological distress at ages 26-42 was calculated using the cross-sectional reporting of the Malaise Index attained at ages 26, 30, 34 and 42.

Back pain at age 46 was self-reported and a cohort member was classified as having back pain if they reported doctor's diagnosed back pain (cohort member reporting if they had visited the doctor for an issue relating to back pain in the past four years or since their last interview) and/or moderate or severe pain (pain severity in the past four weeks measured on a five-point Likert scale). Those reporting moderate or severe pain were assumed to have a condition relating to pain.

Back pain at ages 26-42 was specified using the cross-sectional self-reporting of back pain at ages 26, 30, 34, 38 and 42. We assumed back pain if the cohort member visited a doctor for 'back pain' or 'backache'. At age 42 we additionally included a measure of pain severity, those reporting moderate or severe pain were assumed to have a condition relating to pain. Age 42 was the first sweep at which pain severity was reported.

For peer review only - http://bmjopen.bmj.com/site/about/guidelines.xhtml
For peer review only

Blood pressure at age 46 was classified if the cohort member had measured hypertension or if they had received a doctor's diagnosis of high blood pressure, since diagnosis of high blood pressure may result in a prescription for antihypertensive medication, thus lowering blood pressure readings. Blood pressure was measured in millimeters of mercury and was used to classify individuals as either having hypertension defined as systolic/diastolic BP of at least 140/90 mm Hg, or not,[31].

High blood pressure at ages 30-42 was calculated using the cross-sectional self-reporting of high blood pressure. High blood pressure was not recorded at age 26, although we do not feel this will alter our results as the number reporting high blood pressure by age 26 was likely to be extremely small - less than one percent,[32,33]. At age 30 the cohort member reported if they had ever had high blood pressure and at ages 34, 38 and 42 the cohort member reported if they visited the doctors for a health problem relating to high blood pressure in the last four years or since their last interview.

Statistical analysis:

Descriptive analyses explored the percentage distribution of sociodemographic characteristics for the most common three-condition MLTC-M group compared with the remainder of cohort being considered in this paper. To compare the differences between groups Mann-Whitney U tests were utilised.

Prevalence was calculated by dividing the number of cohort member in the MLTC-M group by the number of cohort members in the BCS70 cohort at age 46. The acquisition sequence of LTCs was established by searching each sweep for onset of each LTC. We tabulated sentinel condition, the number of people reporting a sentinel condition at each sweep and the length of time to accrue all three LTCs after reporting the sentinel condition. The sequence of acquisition of psychological distress, high blood pressure and back pain was established by coding the sweep each LTC was first reported and grouping these into patterns dependent on the order of acquisition of LTCs. The permanency of the reporting of psychological distress was established by deriving the reporting of psychological distress (yes/no) at five sweeps (ages 26, 30, 34, 42 and 46).

We displayed findings using a bubble diagram, bar chart and a Sankey diagram. These were constructed using Excel and using online software package 'jsfiddle',[34]. Analysis of the acquisition of sentinel conditions, the sequence of LTCs and the evaluation of the permanency of the report of psychological distress were conducted using the statistical software package Stata IC 16,[35] and Excel. Mann-Whitney U tests were performed using Stata IC 16.

RESULTS

Multimorbidity cohort characteristics:

Psychological distress was the most common single LTC (n=2835, 33.2%) (Table 1). Back pain and high blood pressure were reported by 2767 (32.4%) and 2010 (23.5%), respectively. The most common three-condition MLTC-M was psychological distress, high blood pressure and back pain reported by 370 (4.3%) (Table 1) of whom 164/370 provided additional information on LTCs at ages 26, 30, 34, 38 and 42. (Figure 1).

For peer review only - http://bmjopen.bmj.com/site/about/guidelines.xhtml

Sociodemographic characteristics at age 46:

At age 46, for those in the most common three-condition MLTC-M group, a larger proportion of participants (24.3%) lived in areas in the lowest IMD quintile compared with the remainder of the BCS70 sample (12.7%) (Table 2). Participants in the MLTC-M group were more likely to report no academic qualifications (38.4% vs 27.7%) and were more likely to be living in social rented accommodation (27.3% vs 9.9%). They were also more likely to Table 1. The frequency of the reporting of long-term conditions at age 46 for the full BCS70 cohort.

		N (%)
One LTC	Psychological distress	2835 (33.2%)
	Back pain	2767 (32.4%)
	High blood pressure	2010 (23.5%)
	Asthma	1003 (11.7%)
	Arthritis	652 (7.6%)
Three LTCs	Psychological distress, back pain and high blood pressure	370 (4.3%)
	Arthritis, psychological distress and back pain	240 (2.8%)
	Asthma, psychological distress and back pain	236 (2.8%)
	Asthma, high blood pressure and psychological distress	145 (1.7%)
	Asthma, high blood pressure and back pain	134 (1.6%)
	Arthritis, high blood pressure and back pain	131 (1.5%)
	Arthritis, high blood pressure and psychological distress	113 (1.3%)
	Arthritis, asthma and back pain	89 (1.0%)
	Arthritis, asthma and psychological distress	84 (1.0%)
	Arthritis, asthma and high blood pressure	50 (0.6%)
	Total	*8536*

For peer review only - http://bmjopen.bmj.com/site/about/guidelines.xhtml

Table 2. Sociodemographic characteristics of MLTC-M group (psychological distress, high blood pressure and back pain), compared with remainder of BCS70 sample at age 46.

		MLTC-M	Non MLTC-M	Mann-Whitney U Tests
		N (%)	N (%)	
Sex				p=0.737
	Male	173 (46.8%)	3953 (48.4%)	
	Female	197 (53.2%)	4213 (51.6%)	
IMD quintiles				**p=0.000**
	1 (most deprived)	90 (24.3%)	1039 (12.7%)	
	2	74 (20%)	1315 (16.1%)	
	3	87 (23.5%)	1701 (20.8%)	
	4	68 (18.4%)	1981 (24.6%)	
	5 (least deprived)	51 (13.8%)	2130 (26.1%)	
Highest academic qualifications				**p=0.000**
	No qualification	142 (38.4%)	2263 (27.7%)	
	GCSE or equivalent	139 (37.6%)	2651 (32.5%)	
	A level or equivalent	42 (11.3%)	1130 (13.9%)	
	Degree or higher	47 (12.7%)	2122 (26.0%)	
Housing tenure				**p=0.000**
	Own/Mortgage	218 (58.9%)	6551 (80.2%)	
	Social rent	101 (27.3%)	812 (9.9%)	
	Private rent/other	51 (13.8%)	803 (9.8%)	
Local authority/housing association				**p=0.000**
	No	269 (72.7%)	7354 (90.1%)	
	Yes	101 (27.3%)	812 (9.9%)	
Employment status				
	Employed	245 (66.2%)	7239 (88.7%)	**p=0.000**
	Unemployed	9 (2.4%)	144 (1.8%)	
	Sickness/disability	72 (19.5%)	271 (3.5%)	
	Caring for family	35 (9.5%)	410 (5.0%)	
	Other	9 (2.4%)	102 (1.3%)	
Benefits (including child benefits)				**p=0.000**
	No	170 (46.0%)	5171 (63.3%)	
	Yes	200 (54.0%)	2995 (36.7%)	
Total		370 (100%)	8166 (100%)	

Significant difference was found using a Mann-Whitney U Test included in bold - excluding missing

For peer review only

receive benefits (54.0% vs 36.7%) and were more likely to be off work for issues relating to sickness (19.5% vs 3.5%). These differences were all statistically significant at the 5% level (Table 2).

Sentinel conditions for the MLTC-M subgroup (n=164):

For the 164 participants with the most common three-condition MLTC-M at age 46 who also provided information on LTCs at ages 26, 30, 34, 38 and 42, psychological distress (n=41, 25.0%), back pain (n=35, 21.3%) and the co-reporting of psychological distress and back pain (n=35, 21.3%) were the three most common sentinel conditions/combinations (Figure 2). Hypertension was the sentinel condition for 15 (9.1%).

At age 26, 74/164 of the subgroup (45.1%), reported the sentinel condition (Figure 2). Most common sentinel conditions at age 26 included back pain, reported by 26 (15.9%), the co-reporting of psychological distress and back pain reported by 25 (15.2%) and the reporting of psychological distress 20 (12.2%).

Sequence of accrual of LTCs for the MLTC-M subgroup (n=164):

For the MLTC-M subgroup (n=164) the most common sequence of LTCs was the co-reporting of psychological distress and back pain followed by high blood pressure (n=35, 21.3%) (Figure 3). Other common sequences included psychological distress followed by back pain and then high blood pressure (n=16, 9.8%) and back pain followed by high blood pressure and then psychological distress (n=15, 9.1%).

The variation in the reporting of psychological distress for the MLTC-M subgroup (n=164):

For the MLTC-M subgroup (n=164), 27 (16.4%) reported psychological distress at age 46 only. 14 (8.5%) reported psychological distress at all sweeps, 31 (18.9%) reported psychological distress at one sweep and then continued to report psychological distress at all subsequent sweeps and 42 (25.6%) reported psychological distress at one sweep and then at age 46 (Figure 4). 35 (21.3%) reported one fluctuation in the reporting of psychological distress (no-yes-no-yes/yes-no-yes) and 15 (9.2%) reported multiple fluctuations in the reporting of psychological distress (no-yes-no-yes-no-yes/yes-no-yes-no-yes) (Figure 4). Further, 29 (17.7%) reported a U shape psychological distress trajectory with elevated levels of psychological distress in their mid-20s and at midlife.

DISCUSSION

Principal findings:

In this national birth cohort, 8536 individuals provided information on LTCs at age 46. 370 (4.3%) people had the most common three-condition MLTC-M grouping - psychological distress, back pain and high blood pressure. Descriptive analysis and Mann-Whitney U tests suggests that those with the three-condition MLTC-M were characterised by worse sociodemographic factors. For the 164 (44.3%) MLTC-M subgroup who reported information on LTCs across adulthood the most common sentinel condition was psychological distress, followed by back pain and the most common sequence of accrual of LTCs was the co-reporting of psychological distress and back pain followed by high blood

For peer review only

pressure. Just under a third of the MLTC-M subgroup (n=50, 30.5%) reported a variation in the reporting of psychological distress across the adult lifecourse.

Comparison with other studies:

Few studies have described the sequences of accrual of LTCs across the lifecourse or explored the sentinel condition. We found psychological distress to be the sentinel condition for a quarter of the sample. This supports research among an urban multi-ethnic borough in London, which found that multimorbidity in those under 65 tended to start with depression or serious mental illness,[4]. Further, among younger cohorts (age 20-39), the most common sequence of accrual of LTCs began with depression,[36]. Our findings are comparable with other studies that have reported the high prevalence of mental ill-health among multimorbidity cohorts,[37,38]. There is therefore increasing evidence emphasising the need to target prevention efforts aimed at mental ill-health in the early lifecourse. Future research could consider what role targeted mental health prevention may play in altering multimorbidity pathways.

Research using longitudinal analysis to report sequencing and clusters of LTCs across the lifecourse is scarce,[4]. An Australian longitudinal study found that in a cohort of women over the age of 50, 17% developed multimorbidity over a 20-year period,[39]. The onset of stroke was most strongly associated with the progression to multimorbidity (23%), whereas a smaller proportion of women progressed to multimorbidity after an initial diagnosis of diabetes (10%) or heart disease (11%),[39]. Our research supports a similar lifecourse trajectory timeline: 22% of the cohort took 16 years to accrue all three LTCs and 20% took 20 years.

A longitudinal study from Australia considered eight LTCs over a 10-year period; onset of hypertension, cardiovascular disease and chronic obstructive pulmonary disease (COPD) were likely to occur at an older age and hypercholesterolemia, asthma and mental health problem at younger ages,[40]. Although hypercholesterolemia was more likely to occur as a first chronic disease (CD), it was significantly associated with a lower risk of developing further CDs,[24]. Alternatively, mood and anxiety disorders or asthma at baseline was associated with the risk of developing further CDs,[40]. Those reporting mood and anxiety disorders were at a higher subsequent risk of diabetes, asthma and mental disorders; those reporting asthma were at a higher risk of developing COPD and hypercholesterolaemia,[40].

Finally, a study from the US explored the acquisition sequence of 20 LTCs describing both two- and three-condition multimorbidity,[36]. The most common sequence of three-condition multimorbidity in 20-39 year olds was depression, asthma and substance misuse, whereas in 50–59 year olds, it was hyperlipidaemia, hypertension and diabetes,[36]. Our research supports a similar sequence of accrual timeline to previous studies,[36,40]. We found psychological distress was likely to be the sentinel condition at younger ages. High blood pressure was infrequently reported as the sentinel condition and likely to be reported at an older age, often as the last condition.

Permanency of the psychological distress over time:

Previous research suggest that mental health trajectories have been found to influence future health outcomes,[13-16]. 45 (27.4%) of the MLTC-M subsample had persistent psychological distress across sweeps and 50 (30.5%) reported psychological distress that

For peer review only - http://bmjopen.bmj.com/site/about/guidelines.xhtml

fluctuated. This represents a substantial proportion of people with transient psychological distress pathways and supports the need to consider how the cross-sectional reporting of LTC may not reflect the variation in certain LTC over the lifecourse. It also demonstrates the need to consider how the recording of a mental health diagnoses as permanent in healthcare records may not reflect the true experience for many individuals. Further, research that has considered psychological distress trajectories found that the BCS70 cohort experienced elevated levels of psychological distress in their mid-20s compared with their early-mid 30s,[24] and that midlife appeared to be a particularly vulnerable phase for experiencing psychological distress,[24]. Our MLTC-M subgroup supported a similar psychological distress lifecourse trajectory.

Meaning of the study:

Among the BCS70 cohort two condition multimorbidity at age 46-48 was common (34%),[19]. Furthermore, almost a third of people in a representative primary care dataset who reported two LTCs were younger than 65 years,[3]. Ruel et al reported a two condition multimorbidity prevalence of 32% with a mean age of 50 years,[37]. Sauver et al concluded that the total number of people who developed multimorbidity before age 65 years was more than four times greater than the number at ages 65 and over,[34]. Our research determined that the most common three-condition MLTC-M in the BCS70 was infrequent (4.3%). However, this still represents a large number of people living with early-onset MLTC-M, and considering all ten three-condition MLTC-M we find a greater proportion of the BCS70 affected. Therefore increasingly conclusive evidence emphasises the need to target multimorbidity prevention efforts at a younger age. This is supported by our findings that 74 (45.1%) of the MLTC-M subgroup reported the sentinel condition by age 26.

Multimorbidity prevalence differs according to demographic factors. Barnett et al found that the most deprived areas had the same prevalence of multimorbidity as people aged 10-15 years older living in the most affluent areas,[38]. Ashworth et al concluded that ethnicity and social deprivation were significant determinants of multimorbidity,[7]. Sauver et al suggested that the incidence of both two- and three-condition multimorbidity varied by age and ethnicity,[36]. Gondek et al also identified socioeconomic circumstances as early life determinants of multimorbidity amongst the BCS70 cohort,[17]. We demonstrate the reporting of the most common three condition MLTC-M was patterned by sociodemographic factors encompassing both area measures (IMD) and individual-level measures of deprivation (employment, education and benefits).

From a public health perspective, describing the sequence of accrual of LTCs, identifying the sentinel condition and exploring the permanency of the reporting of certain LTCs could provide the opportunity to identify critical timepoints for population-level prevention efforts to reduce multimorbidity burden. We plan future work to explore the way in which determinants of multimorbidity may be similar or different from determinants of the sentinel condition and sequence of accrual of LTCs on the trajectory towards multimorbidity. Multimorbidity at midlife is potentially more burdensome, particularly for those of working age. In a cross-sectional study of about 800 people with multimorbidity in the UK, higher treatment burden was associated with people aged 55-64 vs. those over 65,[41]. The sentinel condition is also an indicator of a pathway to future health outcomes and therefore identifying the sentinel condition of multimorbidity is important for targeted preventions with the aim of altering pathways to further health outcomes. This paper has demonstrated the potential of using birth cohort data to study MLTC-M across the lifecourse. Future work may look to

For peer review only - http://bmjopen.bmj.com/site/about/guidelines.xhtml

sequence LTCs and the sentinel condition on a larger scale and we hope this paper can be the foundation for this research.

Strengths and limitations:

The use of a prospective longitudinal approach provides the opportunity to analyse the time-sequenced nature of the data,[42]. Furthermore, there are multiple wider determinant exposures captured in BCS70, unavailable in most other datasets which will provide opportunities for future lifecourse investigation. Knowing the temporal sequencing of LTCs offers valuable means for elucidating an understanding of the accrual sequence of LTCs. However, the BCS70, like other major cohort studies, is affected by significant attrition and wave non-response. This is a limitation of our study – there were few people who reported both the most common three-condition MLTC-M at 46 and information on LTCs across all the adult sweeps (164/370). We therefore risk bias from underreporting of the MLTC-M group. Further, the small sample size also precludes the opportunity to explore men and women independently. However, this paper is exploratory: we do not conduct any regression analysis and are cautious not to overstate our findings. We recommend that future work using formal statistical methods consider accounting for attrition and wave non-response.

Data quality remains an important limitation. The variables used to assess the sequence of accrual of LTCs did not match up identically across sweeps and we were required to make assumptions about whether variables captured the same LTCs. Further, at age 26, blood pressure was not recorded, although the number of those reporting high blood pressure by age 26 was likely to be small,[32,33]. Data coding constraints meant we were unable to provide a more precise measurement of when the sentinel condition was reported or differentiate between the times of onset of conditions if they were first recorded at the same sweep. Further work in birth cohorts to harmonise and agree LTC definitions would strengthen their potential for multimorbidity research.

Some LTCs were self-reported and are therefore likely under-recorded, implying that we may be underestimating the true prevalence of multimorbidity. The cohort is representative of a cohort of children born in 1970, and as such does not reflect the ethnically diverse population of the UK today (Supplementary Materials Table 1). It is known from previous research that the prevalence of multimorbidity is more common and occurs earlier in certain ethnic minority populations,[7,36]. We are also aware that health is not static and we aim to incorporate further methods capturing fluctuation in other LTCs when exploring the sequence of accrual of LTCs. Further, we focus on the relative risk of psychological distress; this measure is limited to symptoms of depression and anxiety, and does not capture the full range of other mental health problems.

Finally, these analyses were exploratory and descriptive. Caution must be taken not to overstate our findings given the limitations of sample size, attrition and the fluctuation in the recording of LTCs. Limitations of these analyses precluded further statistical analysis. Future work should consider multiple wider determinant exposures captured in birth cohort datasets and explore the associations between the early lifecourse and both sentinel conditions and early onset burdensome multimorbidity clusters. However, more careful consideration around the role of confounders, mediators, attrition, sample size and the reporting of LTCs are required.

For peer review only - http://bmjopen.bmj.com/site/about/guidelines.xhtml

CONCLUSIONS

In these exploratory analyses in a national birth cohort, while overall prevalence of the most common three-condition burdensome multimorbidity group was relatively low (4.3%) at age 46, we identified psychological distress and back pain as the most common sentinel conditions. The MLTC-M group was characterised by poorer sociodemographic factors. Birth cohorts may usefully inform lifecourse-focused multimorbidity research but there are current limitations to their utility. We cannot influence the BCS70 sample size and diversity but future work could combine with other birth cohorts that include people into their 60s. Further work is also needed to agree on LTC definitions to strengthen their potential for multimorbidity research.

<u>Footnotes</u>

Author Contributions: SS, EH, NA, MB, RH, ZZ, SC and SF were involved in the conceptualisation of ideas. SF obtained the BCS70 data. SS, EH, NA, and SF designed and planned the analyses. SS and EH performed the statistical analysis. SS initial draft of manuscript. SS, EH, NA, SC, RH, MB, WW, JM and SF were involved in drafting, editing and reviewing the manuscript. All authors contributed to the development of the project and were involved in the approval of the final manuscript. SS and SF take responsibility for the data and research governance.

Funding: This research is supported by NIHR grant number [NIHR202644] as part of a larger research project titled *'Developing a Multidisciplinary Ecosystem to Study Lifecourse Determinants of Complex Mid-life Multimorbidity using Artificial Intelligence (MELD)'*.

Disclaimer: The funder of the study had no role in the study design, data collection, data analysis, data interpretation, or the writing of the paper.

Competing interests: All authors (SS, EH, NA, MB, RH, ZZ, SC, MA, SF, JM and WW) have no competing interests.

Ethical approval: Ethical approval for the BCS70 study was granted by the National Health Service (NHS) Research Ethics Committee, and all participants provided fully informed consent. For details on the BCS70 ethics and consent please see: https://cls.ucl.ac.uk/wp-content/uploads/2017/07/BCS70-ethical-review-and-consent-Shepherd-P-November-2012.pdf. Ethics approval for this analysis was granted by Southampton University Ethics and Research Governance Online (61451) and by The Proportionate Review Sub-committee of the London - Camden & Kings Cross Research Ethics Committee (21/PR/0023).

Provenance and peer review: Not commissioned; externally peer-reviewed.

Data availability statement: The BCS70 datasets generated and analysed in the current study are publicly available in the UK Data Archive repository:
BCS70 https://discover.ukdataservice.ac.uk/series/?sn=200001.

Reference List:

1. Kingston A, Robinson L, Booth H, et al. Projections of multi-morbidity in the older population in England to 2035: estimates from the population ageing and care simulation (PACSim) model. *Age Ageing* 2018;47(3):374–80.

2. Whitty C J M, MacEwen C, Goddard A, et al. Rising to the challenge of multimorbidity. *BMJ* 2020; 368:l6964.

3. Barnett K, Mercer S W, Norbury M, et al. Epidemiology of multimorbidity and implications for health care, research, and medical education: a cross-sectional study. *Lancet.* 2012; 380(9836):37-43.

4. The Academy of Medical Sciences. Multimorbidity: a priority for global health research. London: Academy of Medical Sciences; 2018 [accessed 2021 01/09]. Available from: https://acmedsci.ac.uk/policy/policy-projects/multimorbidity

5. Moussavi S, Chatterji S, Verdes E, et al. Depression, chronic diseases, and decrements in health: Results from the World Health Surveys. *The Lancet*. 2007;370:851–858).

6. Pescosolido B A, Patient trajectories. in The Wiley Blackwell Encyclopedia of Health, Illness, Behavior, and Society, First Edition. Cockerham W C, Dingwall R, Quah SR Eds. 2014 John Wiley & Sons, Ltd.

7. Ashworth M, Durbaba S, Whitney D, et al. Journey to multimorbidity: longitudinal analysis exploring cardiovascular risk factors and sociodemographic determinants in an urban setting. *BMJ Open* 2019;9(12):e031649.

8. Fortin M, Stewart M, Poitras M E, et al. A systematic review of prevalence studies on multimorbidity: toward a more uniform methodology. *Ann Fam Med* 2012;10(2):142–51

9. Mujica-Mota R E, Roberts M, Abel G, et al. Patterns of morbidity and multi-morbidity and their impact on health-related quality of life: evidence from a national survey. *Qual Life Res* 2015;24(4):909–18.

10. Rhebergen D, Batelaan N M, de Graaf R, et al. (2011), The 7-year course of depression and anxiety in the general population. Acta Psychiatrica Scandinavica, 123: 297-306.

11. Gustavson K, Knudsen A K, Nesvåg R, et al. (2018). Prevalence and stability of mental disorders among young adults: Findings from a longitudinal study. *BMC Psychiatry, 18,* Article 65.

12. Lamers F, Beekman A T, van Hemert A M, et al. Six-year longitudinal course and outcomes of subtypes of depression. Br J Psychiatry. 2016;208(1):62–8.

13. Brodaty H, Luscombe G, Peisah C, et al. A 25-year longitudinal, comparison study of the outcome of depression. Psychol Med. 2001;31:1347–1359.

For peer review only

14. Mueller T I, Leon A C, Keller M B, et al. Recurrence after recovery from major depressive disorder during 15 years of observational follow-up. Am J Psychiatry. 1999;156:1000–1006.

15. Roshanaei-Moghaddam B, Katon W J, & Russo J. The longitudinal effects of depression on physical activity. General Hospital Psychiatry, 2009: 31(4), 306–315.

16. Doering LV, Moser DK, Riegel B, et al. Persistent comorbid symptoms of depression and anxiety predict mortality in heart disease. *Int J Cardiol*. 2010;145(2):188-192.

17. Mercer S W, Gunn J, Bower P, et al. Managing patients with mental and physical multimorbidity. *BMJ* 2012;345:e5559

18. Multimorbidity: clinical assessment and management NICE guideline [NG56] Published: 21 September 2016 [accessed 2021 01/09]. Available from https://www.nice.org.uk/guidance/ng56.

19. Gondek D, Bann D, Brown M, et al. Prevalence and early-life determinants of mid-life multimorbidity: evidence from the 1970 British birth cohort. *BMC Public Health* 2021, 1319

20. Elliott J, & Shepherd P. Cohort profile: 1970 British Birth Cohort (BCS70). *Int J Epidemiol* 2006;35(4):836–43.

21. (UCL) Centre For Longitudinal Studies. User guides 2019 [Accessed 2019 01/05]. Available from: https://cls.ucl.ac.uk/data_documentation/?types%5B%5D=user-guides&studies%5B%5D&studies%5B%5D=bcs70-birth-sweep&studies%5B%5D=age-5-sweep&studies%5B%5D=age-10-sweep&studies%5B%5D=age-16-sweep-1970-british-cohort-study&studies%5B%5D=age-26-sweep&studies%5B%5D=age-30-sweep&studies%5B%5D=age-34-sweep&studies%5B%5D=age-38-sweep&studies%5B%5D=age-42-sweep-1970-british-cohort-study&studies%5B%5D=age-46-biomedical-sweep&s=&filter=1

22. UCL – 1970 British Cohort Study [accessed 2021 01/09]. Available from: https://cls.ucl.ac.uk/cls-studies/1970-british-cohort-study/

23. UK Data Service [accessed 2021 05/09] https://ukdataservice.ac.uk//app/uploads/teachingresource1970bcsmalaiseinventory.pdf

24. Gondek D, Bann D, Patalay P, et al. (2021). Psychological distress from early adulthood to early old age: Evidence from the 1946, 1958 and 1970 British birth cohorts. Psychological Medicine, 1-10.

25. Mirowsky J, & Ross C E. (2002). Measurement for a human science. Journal of Health and Social Behavior, 43, 152–170

For peer review only - http://bmjopen.bmj.com/site/about/guidelines.xhtml

26. Ploubidis G B, Sullivan A, Brown M, et al. (2017). Psychological distress in midlife: Evidence from the 1958 and 1970 British birth cohorts. Psychological Medicine, 47, 291–303.

27. Ploubidis G B, McElroy E, & Moreira H C. (2019). A longitudinal examination of the measurement equivalence of mental health assessments in two British birth cohorts. Longitudinal and Life Course Studies, 10, 471–489.

28. McGee R, Williams S, & Silva P A. (1986). An evaluation of the malaise inventory. Journal of Psychosomatic Research, 30, 147–152.

29. Furnham A, & Cheng H. (2015). The stability and change of malaise scores over 27 years: Findings from a nationally representative sample. Personality and Individual Differences, 79, 30–34.

30. Rodgers B, Pickles A, Power C, et al. (1999). Validity of the Malaise Inventory in general population samples. Social Psychiatry and Psychiatric Epidemiology, 34, 333–341

31. Messerli F, Rimoldi S, & Bangalore S. Changing definition of hypertension in guidelines: how innocent a number game? *European Heart Journal*. 2018;39(24): 2241–2242

32. Lewington S, Clarke R, Qizilbash N, et al. Age-specific relevance of usual blood pressure to vascular mortality: a meta-analysis of individual data for one million adults in 61 prospective studies. *Lancet*. 2002;360(9349):1903–13

33. NHS Digital. Health Survey for England: High blood pressure [accessed 2021 10/11]. Available from http://healthsurvey.hscic.gov.uk/data-visualisation/data-visualisation/explore-the-trends/high-blood-pressure.aspx

34. Jsfiddle [accessed 2021 01/09]. Available from: https://jsfiddle.net

35. StataCorp. 2019. *Stata Statistical Software: Release 16*. College Station, TX: StataCorp LLC.

36. St Sauver J L, Boyd C M, Grossardt B R, et al. Risk of developing multimorbidity across all ages in an historical cohort study: differences by sex and ethnicity. *BMJ Open* 2015;5

37. Cassell A, Edwards D, Harshfield A, et al. The epidemiology of multimorbidity in primary care: a retrospective cohort study. *British Journal of General Practice* 2018; 68 (669): e245-e251

38. Barnett K, Mercer S W, Norbury M, et al. Epidemiology of multimorbidity and implications for health care, research, and medical education: a cross-sectional study. *Lancet* 2012; 380(9836):37-43

For peer review only - http://bmjopen.bmj.com/site/about/guidelines.xhtml

39. Xu X, Mishra G D, Dobson A J, et al. Progression of diabetes, heart disease, and stroke multimorbidity in middle-aged women: A 20-year cohort study. *PLOS Medicine* 2018; 15(3): e1002516.

40. Ruel G, Lévesque J F, Stocks N, et al. Understanding the Evolution of Multimorbidity: Evidences from the North West Adelaide Health Longitudinal Study (NWAHS). *PLOS ONE* 2014; 9(5): e9629.

41. Morris J E, Roderick P J, Harris S, et al. Treatment burden for patients with multimorbidity: cross-sectional study with exploration of a single-item measure. *British Journal of General Practice.* 2021; 71 (706): e381-e390.

42. Wadsworth, M. & Bynner, J. A Companion to Life Course Studies The social and historical context of the British birth cohort studies Introduction. *Routledge Advanced Sociology*. 2011;1-18.

For peer review only

For peer review only - http://bmjopen.bmj.com/site/about/guidelines.xhtml

Figure 1. Selection Flow of MLTC-M sample at age 46

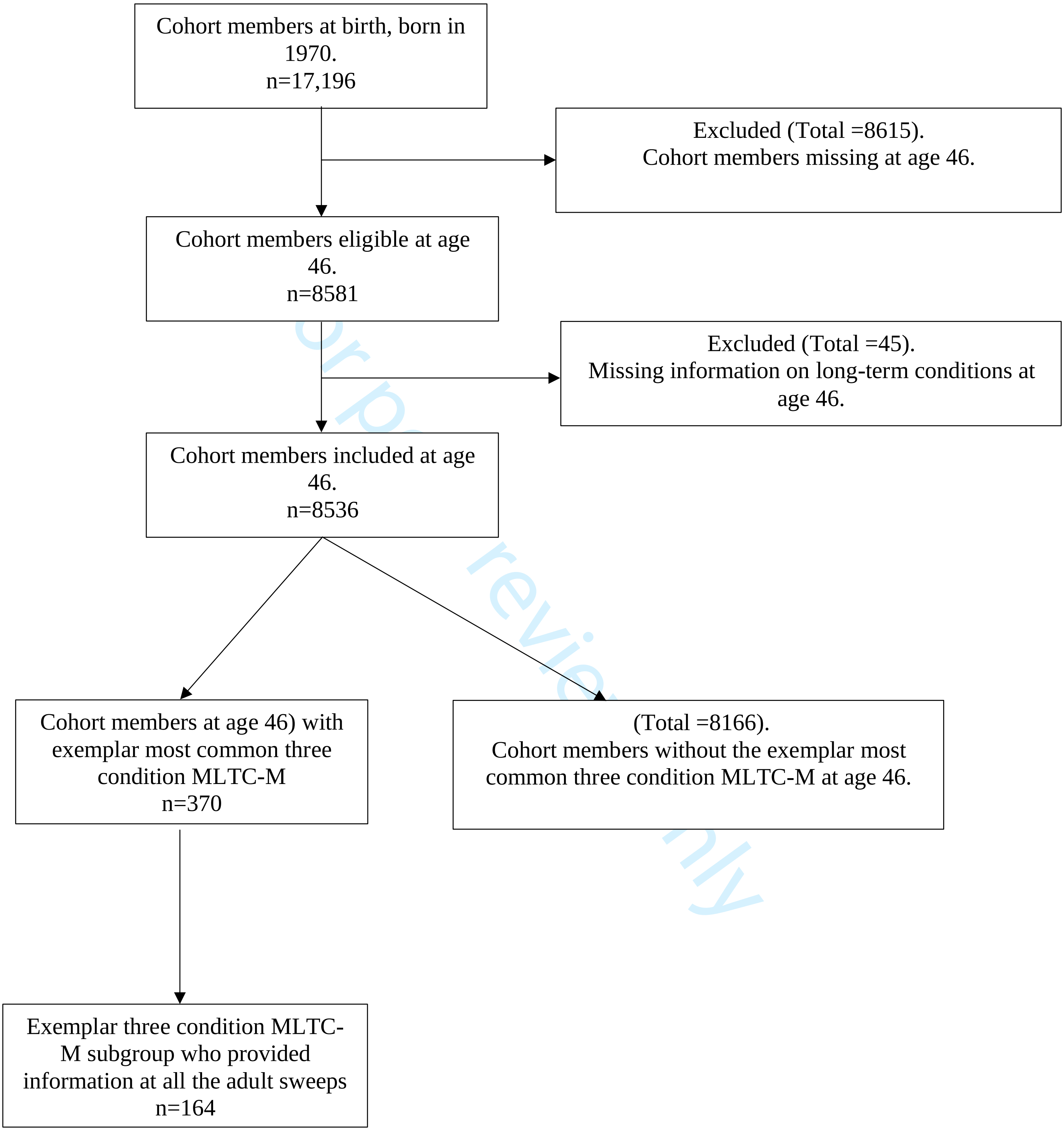

Figure 2. Bubble diagram of the percentage distribution of sentinel conditions and the age at which the sentinel condition was reported, for all 164 cohort members who reported the MLTC-M group of psychological distress, high blood pressure and back pain at age 46 and provided information on LTC across the adult sweeps.

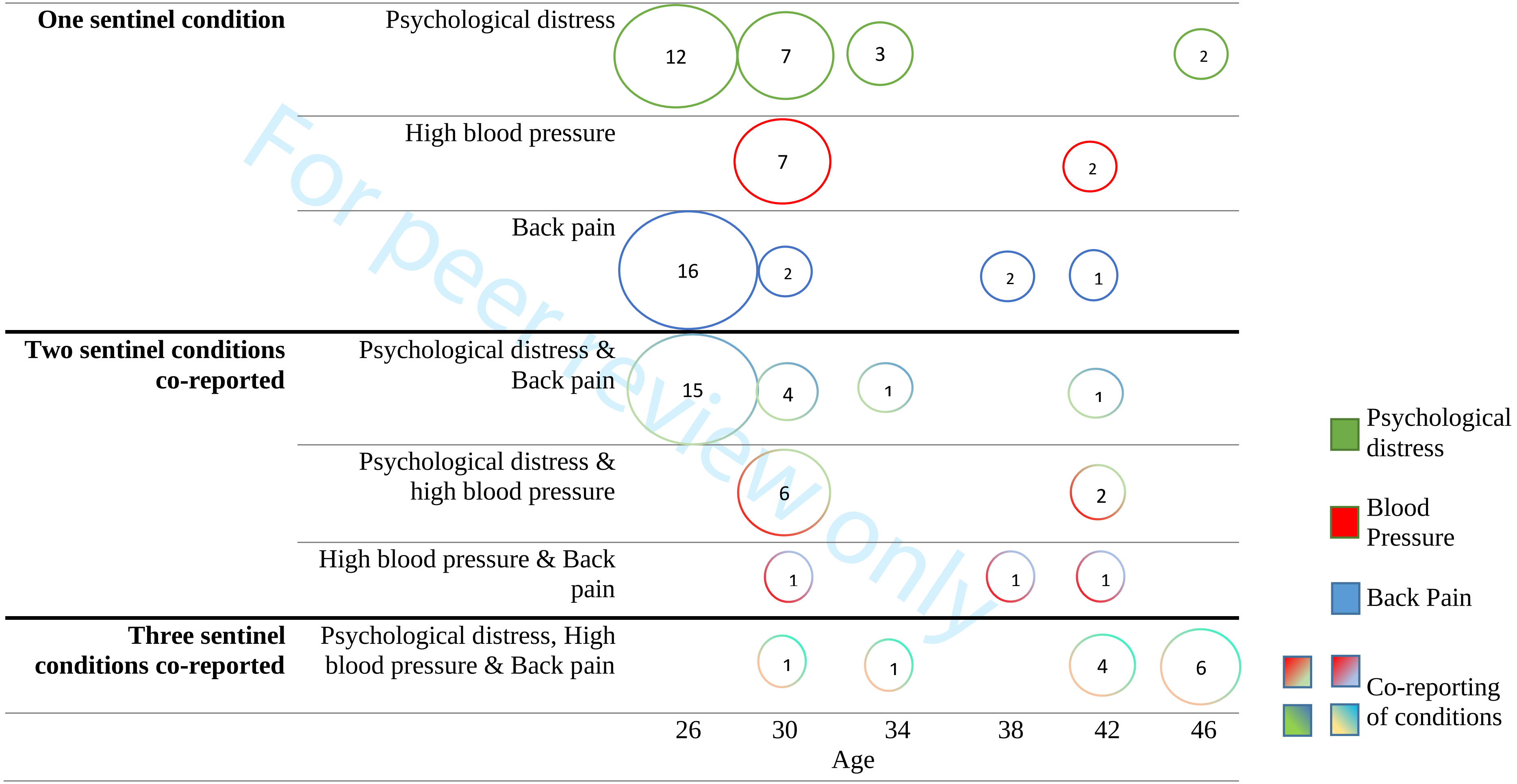

The area of each bubble represents, for those that reported the MLTC-M group of psychological distress, *high blood pressure and back pain at age 46, the reporting of the sentinel condition at each sweep in adulthood. The percentages are included inside each bubble.*

For peer review only

Figure 3. Sankey diagram of sequences of long-term condition for all 164 cohort members who reported the MLTC-M group of psychological distress (PD), high blood pressure (BP) and back pain (pain) at age 46 and provided information on LTC across the adult sweeps.

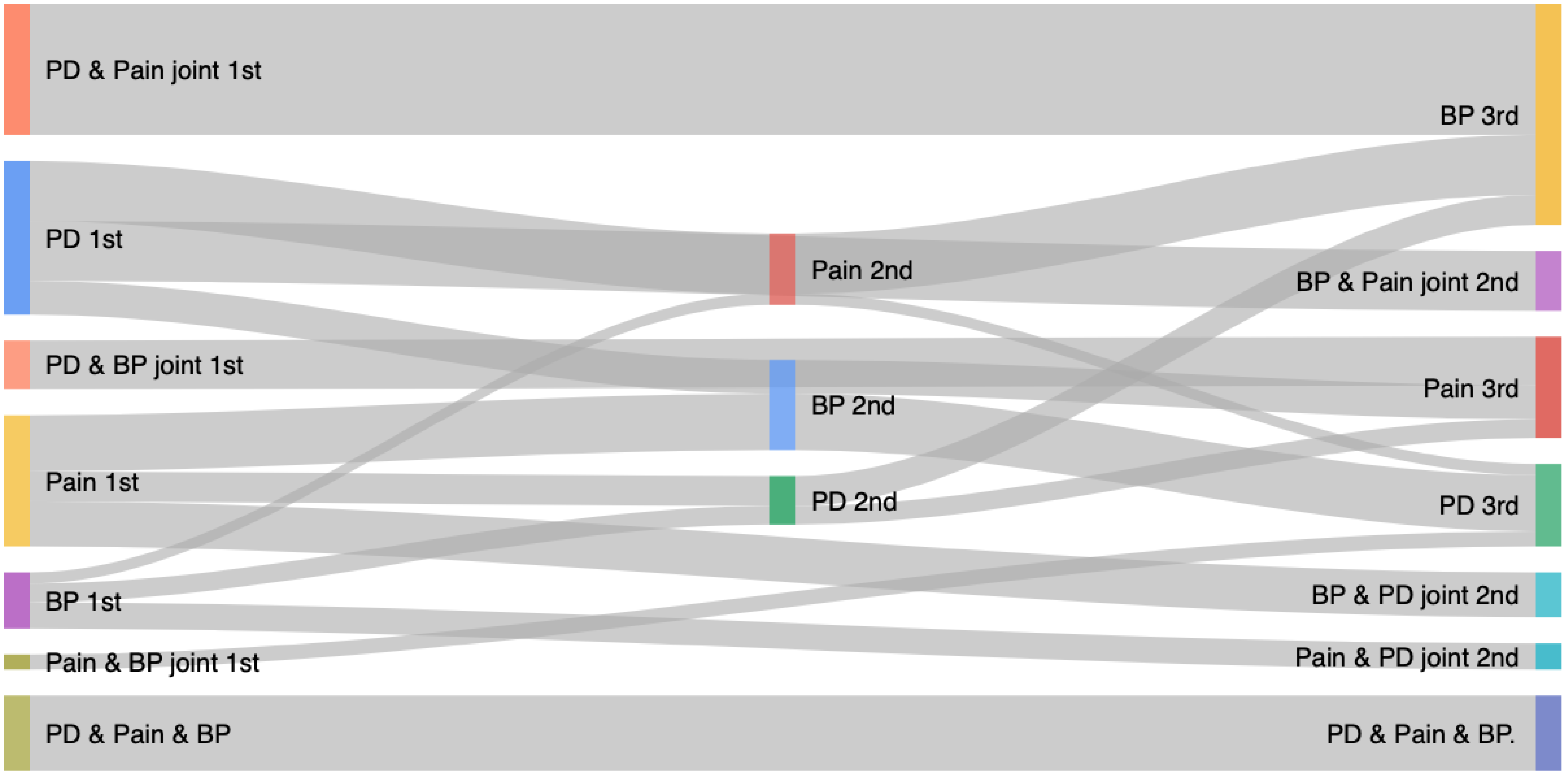

The size of the bar represents the number of people within each pathway. Larger bars representing a greater number of people.

Figure 4. A graph representing the reporting of psychological distress across the adult sweeps for all 164 cohort members who reported the MLTC-M group of psychological distress, high blood pressure and back pain at age 46 and provided information on LTC across the adult sweeps.

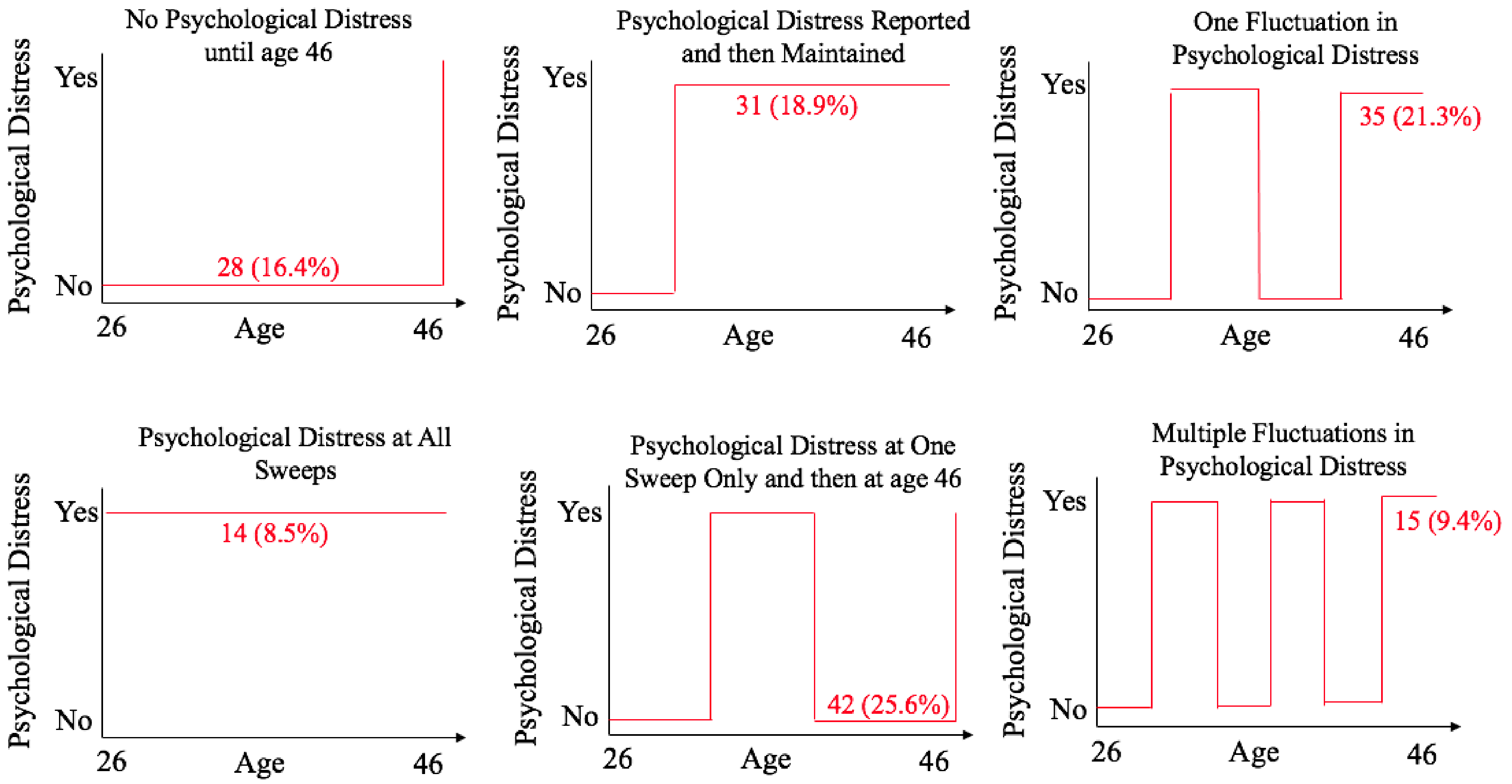

Supplementary Materials

Table 1. Ethnicity of the cohort members at age five[1] for MLTC-M group (psychological distress, high blood pressure and back pain), compared with remainder of BCS70 sample at age 46.

		MLTC-M	Non MLTC-M
		N (%)	N (%)
Ethnicity	White British	276 (94.5%)	6268 (96.7%)
	European	5 (1.7%)	58 (0.9%)
	West Indian	4 (1.4%)	52 (0.8%)
	Indian-Pakistani	5 (1.7%)	77 (1.2%)
	Other Asian	0 (0%)	8 (0.1%)
	African	1 (0.3%)	1 (0.0%)
	Other	1 (0.3%)	16 (0.25%)
	Total	292 (100%)	6480 (100%)

[1]*Ethnicity of the cohort member was not recorded at birth, age 5 was the first sweep where ethnicity was reported.*

For peer review only

Reporting checklist for cohort study.

Based on the STROBE cohort guidelines.

Instructions to authors

Complete this checklist by entering the page numbers from your manuscript where readers will find each of the items listed below.

Your article may not currently address all the items on the checklist. Please modify your text to include the missing information. If you are certain that an item does not apply, please write "n/a" and provide a short explanation.

Upload your completed checklist as an extra file when you submit to a journal.

In your methods section, say that you used the STROBE cohortreporting guidelines, and cite them as:

von Elm E, Altman DG, Egger M, Pocock SJ, Gotzsche PC, Vandenbroucke JP. The Strengthening the Reporting of Observational Studies in Epidemiology (STROBE) Statement: guidelines for reporting observational studies.

		Reporting Item	Page Number
**Title and abstract**			
Title	#1a	Indicate the study's design with a commonly used term in the title or the abstract	1
Abstract	#1b	Provide in the abstract an informative and balanced summary of what was done and what was found	2
**Introduction**			
Background / rationale	#2	Explain the scientific background and rationale for the investigation being reported	3
Objectives	#3	State specific objectives, including any prespecified hypotheses	4
**Methods**			
Study design	#4	Present key elements of study design early in the paper	5

For peer review only

Setting	#5	Describe the setting, locations, and relevant dates, including periods of recruitment, exposure, follow-up, and data collection	5
Eligibility criteria	#6a	Give the eligibility criteria, and the sources and methods of selection of participants. Describe methods of follow-up.	5
Eligibility criteria	#6b	For matched studies, give matching criteria and number of exposed and unexposed	n/a
Variables	#7	Clearly define all outcomes, exposures, predictors, potential confounders, and effect modifiers. Give diagnostic criteria, if applicable	6
Data sources / measurement	#8	For each variable of interest give sources of data and details of methods of assessment (measurement). Describe comparability of assessment methods if there is more than one group. Give information separately for for exposed and unexposed groups if applicable.	5
Bias	#9	Describe any efforts to address potential sources of bias	5
Study size	#10	Explain how the study size was arrived at	5
Quantitative variables	#11	Explain how quantitative variables were handled in the analyses. If applicable, describe which groupings were chosen, and why	5
Statistical methods	#12a	Describe all statistical methods, including those used to control for confounding	
5			
Statistical methods	#12b	Describe any methods used to examine subgroups and interactions	7
Statistical methods	#12c	Explain how missing data were addressed	7
Statistical methods	#12d	If applicable, explain how loss to follow-up was addressed	n/a
Statistical methods	#12e	Describe any sensitivity analyses	
13			

Results

For peer review only

Participants	#13a	Report numbers of individuals at each stage of study—eg numbers potentially eligible, examined for eligibility, confirmed eligible, included in the study, completing follow-up, and analysed. Give information separately for for exposed and unexposed groups if applicable.	7
Participants	#13b	Give reasons for non-participation at each stage	7
Participants	#13c	Consider use of a flow diagram	
7			
Descriptive data	#14a	Give characteristics of study participants (eg demographic, clinical, social) and information on exposures and potential confounders. Give information separately for exposed and unexposed groups if applicable.	7
Descriptive data	#14b	Indicate number of participants with missing data for each variable of interest	
7			
Descriptive data	#14c	Summarise follow-up time (eg, average and total amount)	
7			
Outcome data	#15	Report numbers of outcome events or summary measures over time. Give information separately for exposed and unexposed groups if applicable.	
8			
Main results	#16a	Give unadjusted estimates and, if applicable, confounder-adjusted estimates and their precision (eg, 95% confidence interval). Make clear which confounders were adjusted for and why they were included	9
Main results	#16b	Report category boundaries when continuous variables were categorized	9
Main results	#16c	If relevant, consider translating estimates of relative risk into absolute risk for a meaningful time period	
n/a			

For peer review only

Other analyses	#17	Report other analyses done—eg analyses of subgroups and interactions, and sensitivity analyses	9
**Discussion**			
Key results	#18	Summarise key results with reference to study objectives	12
Limitations	#19	Discuss limitations of the study, taking into account sources of potential bias or imprecision. Discuss both direction and magnitude of any potential bias.	13
Interpretation	#20	Give a cautious overall interpretation considering objectives, limitations, multiplicity of analyses, results from similar studies, and other relevant evidence.	12
Generalisability	#21	Discuss the generalisability (external validity) of the study results	13
**Other Information**			
Funding	#22	Give the source of funding and the role of the funders for the present study and, if applicable, for the original study on which the present article is based	14

The STROBE checklist is distributed under the terms of the Creative Commons Attribution License CC-BY. This checklist was completed on 23. November 2021 using https://www.goodreports.org/, a tool made by the EQUATOR Network in collaboration with Penelope.ai

For peer review only

BMJ Open

Early-onset burdensome multimorbidity: an exploratory analysis of sentinel conditions, condition accrual sequence and duration of three long-term conditions using the 1970 British Cohort Study

Journal:	*BMJ Open*
Manuscript ID	bmjopen-2021-059587.R1
Article Type:	Original research
Date Submitted by the Author:	31-Aug-2022

Complete List of Authors:	Stannard, Sebastian; University of Southampton, School of Primary Care, Population Sciences and Medical Education<br>Holland, Emilia; University of Southampton, School of Primary Care, Population Sciences and Medical Education<br>Crozier, S; University of Southampton Faculty of Medicine, 2. MRC Lifecourse Epidemiology Centre; NIHR, Applied Research Collaboration Wessex<br>Hoyle, Rebecca; University of Southampton, School of Mathematical Sciences<br>Boniface, Michael; University of Southampton, IT Innovation Centre<br>Ahmed, Mazen; University of Southampton, IT Innovation Centre<br>McMahon, James; NIHR, Applied Research Collaboration Wessex<br>Ware, William; NIHR, Applied Research Collaboration Wessex<br>Zlatev, Zlatko; University of Southampton, IT Innovation Centre<br>Alwan, Nisreen; University of Southampton, Academic unit of Primary Care and Population Sciences, Faculty of Medicine; University of Southampton, NIHR Southampton Biomedical Research Centre<br>Fraser, Simon; University of Southampton, School of Primary Care, Population Sciences and Medical Education

**Primary Subject Heading**:	Epidemiology
Secondary Subject Heading:	Public health
Keywords:	EPIDEMIOLOGY, Back pain < ORTHOPAEDIC & TRAUMA SURGERY, Hypertension < CARDIOLOGY, MENTAL HEALTH, PUBLIC HEALTH

SCHOLARONE™
Manuscripts

I, the Submitting Author has the right to grant and does grant on behalf of all authors of the Work (as defined in the below author licence), an exclusive licence and/or a non-exclusive licence for contributions from authors who are: i) UK Crown employees; ii) where BMJ has agreed a CC-BY licence shall apply, and/or iii) in accordance with the terms applicable for US Federal Government officers or employees acting as part of their official duties; on a worldwide, perpetual, irrevocable, royalty-free basis to BMJ Publishing Group Ltd ("BMJ") its licensees and where the relevant Journal is co-owned by BMJ to the co-owners of the Journal, to publish the Work in this journal and any other BMJ products and to exploit all rights, as set out in our licence.

The Submitting Author accepts and understands that any supply made under these terms is made by BMJ to the Submitting Author unless you are acting as an employee on behalf of your employer or a postgraduate student of an affiliated institution which is paying any applicable article publishing charge ("APC") for Open Access articles. Where the Submitting Author wishes to make the Work available on an Open Access basis (and intends to pay the relevant APC), the terms of reuse of such Open Access shall be governed by a Creative Commons licence – details of these licences and which Creative Commons licence will apply to this Work are set out in our licence referred to above.

Other than as permitted in any relevant BMJ Author's Self Archiving Policies, I confirm this Work has not been accepted for publication elsewhere, is not being considered for publication elsewhere and does not duplicate material already published. I confirm all authors consent to publication of this Work and authorise the granting of this licence.

For peer review only

Early-onset burdensome multimorbidity: an exploratory analysis of sentinel conditions, condition accrual sequence and duration of three long-term conditions using the 1970 British Cohort Study

Authors:

Sebastian Stannard[1], Emilia Holland[1], Sarah R Crozier[2,3], Rebecca Hoyle[4], Michael Boniface[5], Mazen Ahmed[5], James McMahon[3], William Ware[3], Zlatko Zlatev[5], Nisreen A Alwan[1,6,3] Simon DS Fraser[1]

1. School of Primary Care, Population Sciences and Medical Education, Faculty of Medicine, University of Southampton, UK
2. MRC Lifecourse Epidemiology Centre, Faculty of Medicine at the University of Southampton, UK
3. NIHR Applied Research Collaboration Wessex, Southampton Science Park, Innovation Centre, 2 Venture Road, Chilworth, Southampton, SO16 7NP
4. School of Mathematical Sciences, University of Southampton, Southampton, UK
5. University of Southampton, IT Innovation Centre, Southampton, UK
6. NIHR Southampton Biomedical Research Centre, University of Southampton and University Hospital Southampton NHS Foundation Trust, Southampton, UK

Corresponding Author:

Sebastian Stannard

Murray Building (58)
59 Salisbury Road
Southampton
SO17 1TW

S.J.Stannard@soton.ac.uk

Word count: 4125

For peer review only

Abstract

Objectives: The prevalence of multiple long-term condition (LTC) multimorbidity is increasing with younger onset amongst socioeconomically deprived populations. Research on lifecourse trajectories towards multimorbidity is limited and early-onset multimorbidity poorly characterised. Understanding sentinel conditions (the first LTC occurring in the lifecourse), the sequence of LTC accrual and the permanency of the reporting of LTCs may help identify time points for prevention efforts. We utilised a longitudinal birth cohort to estimate the prevalence of a common three-condition early-onset multimorbidity (MLTC-M) group at midlife, describe the frequency of sentinel conditions, the sequence of LTC accrual and explore the permanency of one of these conditions: psychological distress.

Setting: 1970 British Cohort Study (BCS70).

Participants: 17,196 cohort members born in 1970.

Outcome measures: Prevalence of the most common three-condition multimorbidity group at age 46. The nature and timing of sentinel conditions, the sequencing patterns of subsequent LTC accrual and the permanency of the reporting of psychological distress.

Results: At age 46 high blood pressure, psychological distress and back pain were the most common three-condition MLTC-M group, (4.3%,n=370). A subgroup of 164 (44.3%) people provided complete information on LTC across all timepoints. Psychological distress measured by the Malaise Index was the most common sentinel condition, occurring in 25.0% (n=41), followed by back pain (22%,n=36). At age 26, 45.1% (75/164) reported their sentinel condition. The most common sequence of LTC accrual was the co-reporting of psychological distress and back pain followed by high blood pressure. Almost a third (30.5%,n=50) reported a variation of psychological distress across the adult lifecourse.

Conclusion: In these exploratory analyses, psychological distress and back pain were the most common sentinel conditions, and along with high blood pressure these three conditions represented the most common three-condition MLTC-M group. These analyse suggest that birth cohorts, like the BCS70, may usefully inform lifecourse- multimorbidity research.

Keywords: multimorbidity, long-term conditions, lifecourse, early-onset, birth cohort

Strengths and Limitations of this study:

- The use of a prospective longitudinal approach provides the opportunity to analyse the time-sequenced nature of the data.
- Knowing the temporal sequencing of long-term conditions enabled us to describe the frequency of sentinel conditions, the sequence of long-term condition accrual and explore the permanency of the reporting of a long-term condition.
- Using birth cohort data, with repeated validated measures, allowed us to explore and characterise sequences that incorporated the concept of 'burdensome' multimorbidity by combining both mental and physical conditions.
- The study was limited by sample size, wave-non response and no agreed definition of long-term condition.
- The self-reporting of long-term conditions and the lack of ethnic diversity resulted in the probable underreporting of multimorbidity.

For peer review only - http://bmjopen.bmj.com/site/about/guidelines.xhtml

INTRODUCTION

In the UK, the prevalence of multiple long-term condition multimorbidity (MLTC-M) has increased over the last two decades and is projected to continue to rise,[1]. This presents a significant challenge to health and prevention services,[2].

MLTC-M occurs earlier in the lifecourse among people from more socioeconomically disadvantaged backgrounds,[3]. While often considered a condition of older age, the majority of people living with multimorbidity are under 65,[3]. Earlier and greater accrual of long-term conditions (LTCs) may be influenced by the timing and nature of the exposure to key risk factors or other LTCs and the influence of wider determinants at different life stages.

Research on MLTC-M occurring by midlife remains limited,[4]. Growing evidence suggests that the sequence of accrual of LTCs varies considerably and influences outcomes,[5]. The sentinel condition (the first long-term condition occurring in the lifecourse in the development of MLTC-M), may play an important role. Sentinel conditions may significantly influence subsequent behaviour and other factors that influence the eventual cluster of LTCs, in turn influencing the nature and risk of important outcomes. Diagnosis of a condition is associated with actions such as medication prescription, specialist referral, and self-management advice, that may alter future trajectories of risk. For example, hypertension diagnosis leading to antihypertensive prescription and physical activity advice reducing future stroke risk. The time point of diagnosis therefore becomes an important determinant of future events,[6].

The sequence of LTC development may also be a key determinant of the nature of eventual LTC clusters,[7]. In a longitudinal analysis using UK primary care data from London, Ashworth et al identified that multimorbidity in those under 65 tended to start with depression or serious mental illness followed by combinations of obesity, chronic pain and diabetes,[7]. In contrast, for those aged 65 and over, diabetes and coronary heart disease were the predominant sentinel conditions, followed by combinations of chronic kidney disease, heart failure and stroke,[7]. Additionally, the prevalence of multimorbidity tends to rise steeply in midlife,[8] and middle-aged people with multimorbidity tend to have poorer health related quality of life,[9].

Across the lifecourse, both physical and mental LTCs may not be permanent and may be of variable severity. For example, mental health conditions such as anxiety and depression are often not experienced as a 'fixed state',[10-12]. However, those with a persistent and chronic trajectory of depression tend to have poorer outcomes including suicide, a decrease in physical activity, ischemic heart disease and mortality,[13-16]. Using birth cohort data, with repeated validated measures, affords the opportunity to explore mental health trajectories across the lifecourse. Understanding the patterns of the reporting of mental health for those with MLTC-M may be important for identifying high-risk temporal life periods in the development of multimorbidity.

There also remains limited research on the differential burden experienced by patients with different multimorbidity clusters. Despite research suggesting the relationship between mental and physical health conditions is bidirectional,[17], there remains a need to understand what constitutes burden and complexity for people with multimorbidity. In particular, the National Institute for Health and Care Excellence multimorbidity guidelines

For peer review only - http://bmjopen.bmj.com/site/about/guidelines.xhtml

recommend establishing better understanding of disease burden encompassing both complex disease and combined mental and physical health,[18].

Previous studies have used limited time frames and limited analyses of the sequence of accrual of LTCs,[4]. Using birth cohort data to study multimorbidity at midlife is under-explored. Gondek et al used data from the 1970 British Cohort Study (BCS70) suggesting that multimorbidity (two LTCs) is common at age 46-48 (34%),[19]. Building on this work, we aimed to study the most common three-condition multimorbidity group at age 46 using the BCS70 in order to explore and characterise sequences and incorporate the concept of 'burdensome' multimorbidity by combining both mental and physical conditions. We aimed to describe the nature, timing and recurrence of the sentinel condition for those reporting the most common three-condition MLTC-M, to illustrate the sequence of accrual of LTCs following the sentinel condition and describe the permanency of the reporting of psychological distress.

METHODS

Birth cohort study:

The BCS70 followed 17,096 participants born in 1970 from across England, Scotland and Wales. Since birth, data was collected in 'sweeps' roughly every four years resulting in 10 sweeps of data collection to date. The BCS70 datasets generated and analysed in the study are available in the UK Data Service repository: BCS70,[20], accessed via an End User License Agreement. Extensive information was collected on topics including socio-economic circumstances, family background, cognitive development, education, employment, partnerships, fertility, health and health-related behaviour,[21]. Data was collected using a range of survey methods including self-reported and measured outcomes. A user guide for the dataset can be found at the Centre for Longitudinal Studies,[22]. A cohort profile provides information about the background to the study and full survey methods for each sweep,[21,23].

Study sample:

Information was collected on 17,196 babies born in 1970. At age 46, 8581 (49.9%) of the original population at birth responded - 8536 provided information on LTCs. To derive the sentinel condition and accrual sequence we considered the MLTC-M group at age 46 who provided information across all the sweeps of data collection in adulthood (age 26, 30, 34, 38, 42 and 46) (n=164/370).

Patient and public involvement statement:

Patient and Public Involvement and Engagement (PPIE) co-investigators have been involved at every stage of the research process. Their lived experiences were important for informing the design of the research, incorporation the views of others and shaping our ideas. In particular PPIE input directed us to consider multimorbidity clusters that were both burdensome and complex and to consider the permanency of the reporting of psychological distress.

Variables:

For peer review only - http://bmjopen.bmj.com/site/about/guidelines.xhtml

Sociodemographic measures at age 46:

A number of area-level and individual-level sociodemographic variables were recorded at age 46. Measures included sex (men/women), the Index of Multiple Deprivation (IMD) (in quintiles) and highest academic qualification (no qualification, GCSE or equivalent, A levels or equivalent or degree and higher). Other measures included a three-measure index of housing tenure (own/mortgage, social rent and private rent/other), measure of local authority or housing association housing (yes/no), employment status (employed, unemployed, sickness/disability or caring for family) and a measure of receipt of benefits (yes/no).

Selecting MLTC-M outcome at age 46:

We selected psychological distress, back pain and high blood pressure as our key MLTC-M group at age 46 because our scoping work had suggested that this combination of three LTCs was among the most common MLTC-M group,[24]. The other nine three-condition MLTC-M groups encompassed the most common LTCs at age 46, including psychological distress, back pain, high blood pressure, asthma and arthritis. Psychological distress measured via the Malaise Index is a term developed by the UK data service,[25], and used widely within the context of the British birth cohort studies. Although, psychological distress is not per se pathological and therefore not strictly morbidity but we use it an indicator of mental health. The common three-condition MLTC-M group selected also explored the concept of 'burdensome' multimorbidity by combining both mental and physical conditions.

Long term conditions at ages 26, 30, 34, 38 and 42:

To explore the sentinel condition, sequence of accrual and the permanency of the reporting of psychological distress we required information on LTCs across all of the sweeps in adulthood (age 26, 30, 34, 38 and 42). All measures collected between ages 26 and 42 were self-reported and we had no measured blood pressure. If two or more LTCs were recorded at the same sweep we were unable to identify which condition occurred first.

Psychological distress and anxiety or depression at age 46 was classified if a cohort member had measured high on the Malaise Index,[25] or if they had visited a doctor or specialist for anxiety and/or depression in the last four years or since their last interview. The Malaise Index was selected because it was consistently present at sweeps in adulthood. The Malaise Index was self-reported and designed to capture general signs of psychological distress in adulthood including depression and anxiety symptoms,[26,27]. Scores above the cut off (4 or higher) represent a clinical diagnosis of common mental disorder,[26]. Scalar invariance of the measure was found within and across two birth cohort studies, as well as between genders,[28,29]. The Malaise Index has good psychometric properties,[30], has a good level of internal consistency and its validity is robust across different populations including the general population and high-risk groups,[31,32].

Psychological distress at ages 26-42 was calculated using the cross-sectional reporting of the Malaise Index attained at ages 26, 30, 34 and 42, with scores above the cut off (4 or higher) representing a clinical diagnosis of a common mental disorder,[26]

Back pain at age 46 was self-reported and a cohort member was classified as having back pain if they reported doctor's diagnosed back pain (cohort member reporting if they had visited the doctor for an issue relating to back pain in the past four years or since their last

For peer review only - http://bmjopen.bmj.com/site/about/guidelines.xhtml

interview) and/or moderate or severe pain (pain severity in the past four weeks measured on a five-point Likert scale). Those reporting moderate or severe pain were assumed to have a condition relating to pain.

Back pain at ages 26-42 was specified using the cross-sectional self-reporting of back pain at ages 26, 30, 34, 38 and 42. We assumed back pain if the cohort member visited a doctor for 'back pain' or 'backache'. At age 42 we additionally included a measure of pain severity, those reporting moderate or severe pain were assumed to have a condition relating to pain. Age 42 was the first sweep at which pain severity was reported.

Blood pressure at age 46 was classified if the cohort member had measured hypertension or if they had received a doctor's diagnosis of high blood pressure, since diagnosis of high blood pressure may result in a prescription for antihypertensive medication, thus lowering blood pressure readings. Blood pressure was measured in millimeters of mercury and was used to classify individuals as either having hypertension defined as systolic/diastolic BP of at least 140/90 mm Hg, or not,[33].

High blood pressure at ages 30-42 was calculated using the cross-sectional self-reporting of high blood pressure. High blood pressure was not recorded at age 26, although we do not feel this will alter our results as the number reporting high blood pressure by age 26 was likely to be extremely small - less than one percent,[34,35]. At age 30 the cohort member reported if they had ever had high blood pressure and at ages 34, 38 and 42 the cohort member reported if they visited the doctors for a health problem relating to high blood pressure in the last four years or since their last interview.

Statistical analysis:

Descriptive analyses explored the percentage distribution of sociodemographic characteristics for the most common three-condition MLTC-M group compared with the remainder of cohort being considered in this paper. To compare the differences between groups Mann-Whitney U tests were utilised.

Prevalence was calculated by dividing the number of cohort member in the MLTC-M group by the number of cohort members in the BCS70 cohort at age 46. The acquisition sequence of LTCs was established by searching each sweep for onset of each LTC. We tabulated sentinel condition, the number of people reporting a sentinel condition at each sweep and the length of time to accrue all three LTCs after reporting the sentinel condition. The sequence of acquisition of psychological distress, high blood pressure and back pain was established by coding the sweep each LTC was first reported and grouping these into patterns dependent on the order of acquisition of LTCs. The permanency of the reporting of psychological distress was established by deriving the reporting of psychological distress (yes/no) at five sweeps (ages 26, 30, 34, 42 and 46).

We displayed findings using a bubble diagram, bar chart and a Sankey diagram. These were constructed using Excel and using online software package 'jsfiddle',[36]. Analysis of the acquisition of sentinel conditions, the sequence of LTCs and the evaluation of the permanency of the report of psychological distress were conducted using the statistical software package Stata IC 16,[37] and Excel. Mann-Whitney U tests were performed using Stata IC 16.

For peer review only - http://bmjopen.bmj.com/site/about/guidelines.xhtml

RESULTS

Multimorbidity cohort characteristics:

Psychological distress was the most common single LTC (33.2%,n=2835) (Table 1). Back pain and high blood pressure were reported by 32.4% (n=2767) and 23.5% (n=2010), respectively. The most common three-condition MLTC-M was psychological distress, high blood pressure and back pain reported by 4.3% (n=370) (Table 1) of whom 164/370 provided additional information on LTCs at ages 26, 30, 34, 38 and 42. (Figure 1).

Sociodemographic characteristics at age 46:

At age 46, for those in the most common three-condition MLTC-M group, a larger proportion of participants (24.3%) lived in areas in the lowest IMD quintile compared with the remainder of the BCS70 sample (12.7%) ($p<0.001$) (Table 2). Participants in the MLTC-M group were more likely to report no academic qualifications (38.4% vs 27.7%,$p<0.001$) and were more likely to be living in social rented accommodation (27.3% vs 9.9%,$p<0.001$) compared with the remainder of the BCS70 sample. They were also more likely to receive benefits (54.0% vs 36.7%,$p<0.001$) and were more likely to be off work for issues relating to sickness (19.5% vs 3.5%,$p<0.001$) compared with the remainder of the BCS70 sample.

Table 1. The frequency of the reporting of long-term conditions at age 46 for the full BCS70 cohort.

		N (%)
One LTC	Psychological distress	2835 (33.2%)
	Back pain	2767 (32.4%)
	High blood pressure	2010 (23.5%)
	Asthma	1003 (11.7%)
	Arthritis	652 (7.6%)
Three LTCs	Psychological distress, back pain and high blood pressure	370 (4.3%)
	Arthritis, psychological distress and back pain	240 (2.8%)
	Asthma, psychological distress and back pain	236 (2.8%)
	Asthma, high blood pressure and psychological distress	145 (1.7%)
	Asthma, high blood pressure and back pain	134 (1.6%)
	Arthritis, high blood pressure and back pain	131 (1.5%)
	Arthritis, high blood pressure and psychological distress	113 (1.3%)

For peer review only - http://bmjopen.bmj.com/site/about/guidelines.xhtml

	Arthritis, asthma and back pain	89 (1.0%)
	Arthritis, asthma and psychological distress	84 (1.0%)
	Arthritis, asthma and high blood pressure	50 (0.6%)
	Total	*8536*

Table 2. Sociodemographic characteristics of MLTC-M group (psychological distress, high blood pressure and back pain), compared with remainder of BCS70 sample at age 46.

		MLTC-M	Non MLTC-M	Mann-Whitney U Tests
		N (%)	N (%)	
Sex				p=0.737
	Male	173 (46.8%)	3953 (48.4%)	
	Female	197 (53.2%)	4213 (51.6%)	
IMD quintiles				**p<0.001**
	1 (most deprived)	90 (24.3%)	1039 (12.7%)	
	2	74 (20%)	1315 (16.1%)	
	3	87 (23.5%)	1701 (20.8%)	
	4	68 (18.4%)	1981 (24.6%)	
	5 (least deprived)	51 (13.8%)	2130 (26.1%)	
Highest academic qualifications				**p<0.001**
	No qualification	142 (38.4%)	2263 (27.7%)	
	GCSE or equivalent	139 (37.6%)	2651 (32.5%)	
	A level or equivalent	42 (11.3%)	1130 (13.9%)	
	Degree or higher	47 (12.7%)	2122 (26.0%)	
Housing tenure				**p<0.001**
	Own/Mortgage	218 (58.9%)	6551 (80.2%)	
	Social rent	101 (27.3%)	812 (9.9%)	
	Private rent/other	51 (13.8%)	803 (9.8%)	
Local authority/housing association				**p<0.001**
	No	269 (72.7%)	7354 (90.1%)	
	Yes	101 (27.3%)	812 (9.9%)	
Employment status				
	Employed	245 (66.2%)	7239 (88.7%)	**p<0.001**
	Unemployed	9 (2.4%)	144 (1.8%)	
	Sickness/disability	72 (19.5%)	271 (3.5%)	
	Caring for family	35 (9.5%)	410 (5.0%)	
	Other	9 (2.4%)	102 (1.3%)	
Benefits (including child benefits)				**p<0.001**
	No	170 (46.0%)	5171 (63.3%)	
	Yes	200 (54.0%)	2995 (36.7%)	

For peer review only

For peer review only - http://bmjopen.bmj.com/site/about/guidelines.xhtml

Total		370 (100%)	8166 (100%)	

Significant difference was found using a Mann-Whitney U Test included in bold - excluding missing

Sentinel conditions for the MLTC-M subgroup (n=164):

For the 164 participants with the most common three-condition MLTC-M at age 46 who also provided information on LTCs at ages 26, 30, 34, 38 and 42, psychological distress (25.0%, n=41), back pain (21.3%,n=35) and the co-reporting of psychological distress and back pain (21.3%,n=35) were the three most common sentinel conditions/combinations (Figure 2). Hypertension was the sentinel condition for 9.1% (n=15).

At age 26, 45.1% of the subgroup (n=74/164), reported the sentinel condition (Figure 2). Most common sentinel conditions at age 26 included back pain, reported by 15.9% (n=26), the co-reporting of psychological distress and back pain reported by 15.2% (n=25) and the reporting of psychological distress 12.2% n=20.

Sequence of accrual of LTCs for the MLTC-M subgroup (n=164):

For the MLTC-M subgroup (n=164) the most common sequence of LTCs was the co-reporting of psychological distress and back pain followed by high blood pressure (21.3%,n=35) (Figure 3). Other common sequences included psychological distress followed by back pain and then high blood pressure (9.8%,n=16) and back pain followed by high blood pressure and then psychological distress (9.1%,n=15).

The variation in the reporting of psychological distress for the MLTC-M subgroup (n=164):

For the MLTC-M subgroup (n=164), 16.4% (n=27) reported psychological distress at age 46 only. 8.5% (n=14) reported psychological distress at all sweeps, 18.9% (n=31) reported psychological distress at one sweep and then continued to report psychological distress at all subsequent sweeps and 25.6% (n=42) reported psychological distress at one sweep only and then at age 46 (Figure 4). 21.3% (n=35) reported one fluctuation in the reporting of psychological distress, this group reported psychological distress at one sweep, then did not report psychological distress at a following sweep and then reported psychological distress again at all further sweeps (no-yes-no-yes/yes-no-yes). 9.2% (n=15) reported multiple fluctuations in the reporting of psychological distress; this group reported psychological distress at one sweep, then did not report psychological distress at a following sweep, then reported psychological distress again at the next sweep and then did not report psychological distress at a following sweep, and finally reported psychological distress at age 46 (no-yes-no-yes-no-yes/yes-no-yes-no-yes) (Figure 4). Finally, 17.7% (n=29) reported a U shape psychological distress trajectory with elevated levels of psychological distress in their mid-20s and at midlife.

DISCUSSION

Principal findings:

In this national birth cohort, 8536 individuals provided information on LTCs at age 46. 4.3% (n=370) people had the most common three-condition MLTC-M grouping - psychological distress, back pain and high blood pressure. Descriptive analysis and Mann-Whitney U tests

For peer review only - http://bmjopen.bmj.com/site/about/guidelines.xhtml

suggests that those with the three-condition MLTC-M were characterised by worse sociodemographic factors. For the 44.3% (n=164) MLTC-M subgroup who reported information on LTCs across adulthood the most common sentinel condition was psychological distress, followed by back pain and the most common sequence of accrual of LTCs was the co-reporting of psychological distress and back pain followed by high blood pressure. Just under a third of the MLTC-M subgroup (30.5%,n=50) reported a variation in the reporting of psychological distress across the adult lifecourse.

Comparison with other studies:

Few studies have described the sequences of accrual of LTCs across the lifecourse or explored the sentinel condition. In terms of the sequence of LTC accrual it is of note that as demonstrated in Figure 3, psychological distress on its own or co-reported alongside another condition was a common sentinel condition, and various pathways to multimorbidity emerged from early psychological distress, this is worthy of further exploration in larger datasets with additional long-term conditions. Our findings support research among an urban multi-ethnic borough in London, which found that multimorbidity in those under 65 tended to start with depression or serious mental illness,[7]. Further, among younger cohorts (age 20-39), the most common sequence of accrual of LTCs began with depression,[38]. Our findings are comparable with other studies that have reported the high prevalence of mental ill-health among multimorbidity cohorts,[39,40]. There is therefore increasing evidence emphasising the need to target prevention efforts aimed at mental ill-health in the early lifecourse. Future research could consider what role targeted mental health prevention may play in altering multimorbidity pathways.

Research using longitudinal analysis to report sequencing and clusters of LTCs across the lifecourse is scarce,[4]. An Australian longitudinal study found that in a cohort of women over the age of 50, 17% developed multimorbidity over a 20-year period,[41]. The onset of stroke was most strongly associated with the progression to multimorbidity (23%), whereas a smaller proportion of women progressed to multimorbidity after an initial diagnosis of diabetes (10%) or heart disease (11%),[41]. Our research supports a similar lifecourse trajectory timeline: 22% of the cohort took 16 years to accrue all three LTCs and 20% took 20 years. A longitudinal study from Australia considered eight LTCs over a 10-year period; onset of hypertension, cardiovascular disease and chronic obstructive pulmonary disease (COPD) were likely to occur at an older age and hypercholesterolemia, asthma and mental health problem at younger ages,[42]. Although hypercholesterolemia was more likely to occur as a first chronic disease (CD), it was significantly associated with a lower risk of developing further CDs,[26]. Alternatively, mood and anxiety disorders or asthma at baseline was associated with the risk of developing further CDs,[42]. Those reporting mood and anxiety disorders were at a higher subsequent risk of diabetes, asthma and mental disorders; those reporting asthma were at a higher risk of developing COPD and hypercholesterolaemia,[42].

Finally, a study from the US explored the acquisition sequence of 20 LTCs describing both two- and three-condition multimorbidity,[38]. The most common sequence of three-condition multimorbidity in 20-39 year olds was depression, asthma and substance misuse, whereas in 50–59 year olds, it was hyperlipidaemia, hypertension and diabetes,[38]. Our research supports a similar sequence of accrual timeline to previous studies,[38,42]. We found psychological distress was likely to be the sentinel condition at younger ages. High blood

For peer review only - http://bmjopen.bmj.com/site/about/guidelines.xhtml

pressure was infrequently reported as the sentinel condition and likely to be reported at an older age, often as the last condition.

Permanency of the psychological distress over time:

Previous research suggest that mental health trajectories have been found to influence future health outcomes,[13-16]. 27.4% (n=45) of the MLTC-M subsample had persistent psychological distress across sweeps and 30.5% (n=50) reported psychological distress that fluctuated. This represents a substantial proportion of people with transient psychological distress pathways and supports the need to consider how the cross-sectional reporting of LTC may not reflect the variation in certain LTC over the lifecourse. We demonstrate that the lifecourse pathways to the three condition MLTC-M we considered fluctuated across the population, and use of cross sectional methods would not have identified these diverse pathways to multimorbidity. Our results are also distinct from studies using clinical coding in routine data, where the presence of a diagnosis code often leads to a suggestion of permanency of a diagnosis within health care records. Using a validated tool for assessing psychological distress as we have in this study, rather than a purely self-reported measure, demonstrates the greater complexity of trajectories than might clinic coding. For example over 55% (n=93) of the MLTC-M subsample report a psychological distress trajectory that changed at least once across the adult lifecourse. Previous research that has considered psychological distress trajectories found that the BCS70 cohort experienced elevated levels of psychological distress in their mid-20s compared with their early-mid 30s,[26] and that midlife appeared to be a particularly vulnerable phase for experiencing psychological distress,[26]. Our MLTC-M subgroup showed a similar psychological distress lifecourse trajectory. It is also noteworthy that while we focus on the permanency of psychological distress, other studies have explored the permanency of physical health conditions including asthma,[43], pain,[44] and obesity,[45], and this is worthy of further consideration in future research.

Meaning of the study:

Among the BCS70 cohort two condition multimorbidity at age 46-48 was common (34%),[19]. Furthermore, almost a third of people in a representative primary care dataset who reported two LTCs were younger than 65 years,[3]. Ruel et al reported a two condition multimorbidity prevalence of 32% with a mean age of 50 years,[42]. Sauver et al concluded that the total number of people who developed multimorbidity before age 65 years was more than four times greater than the number at ages 65 and over,[38]. Our research determined that the most common three-condition MLTC-M in the BCS70 was infrequent (4.3%). However, this still represents a large number of people living with early-onset MLTC-M, and considering all ten three-condition MLTC-M we find 18.6% of the BCS70 affected. Therefore increasingly conclusive evidence emphasises the need to target multimorbidity prevention efforts at a younger age. This is supported by our findings that 45.1% (n=74) of the MLTC-M subgroup reported the sentinel condition by age 26.

Multimorbidity prevalence differs according to demographic factors. Barnett et al found that the most deprived areas had the same prevalence of multimorbidity as people aged 10-15 years older living in the most affluent areas,[40]. Ashworth et al concluded that ethnicity and social deprivation were significant determinants of multimorbidity,[7]. Sauver et al suggested that the incidence of both two- and three-condition multimorbidity varied by age and ethnicity,[38]. Gondek et al also identified socioeconomic circumstances as early life

For peer review only

determinants of multimorbidity amongst the BCS70 cohort,[17]. We demonstrate the reporting of the most common three condition MLTC-M was patterned by sociodemographic factors encompassing both area measures (IMD) and individual-level measures of deprivation (employment, education and benefits).

From a public health perspective, describing the sequence of accrual of LTCs, identifying the sentinel condition and exploring the permanency of the reporting of certain LTCs could provide the opportunity to identify critical timepoints for population-level prevention efforts to reduce multimorbidity burden. We plan future work to explore the way in which determinants of multimorbidity may be similar or different from determinants of the sentinel condition and sequence of accrual of LTCs on the trajectory towards multimorbidity. Multimorbidity at midlife is potentially more burdensome, particularly for those of working age. In a cross-sectional study of about 800 people with multimorbidity in the UK, higher treatment burden was associated with people aged 55-64 vs. those over 65,[46]. The sentinel condition is also an indicator of a pathway to future health outcomes and therefore identifying the sentinel condition of multimorbidity is important for targeted preventions with the aim of altering pathways to further health outcomes. This paper has demonstrated the potential of using birth cohort data, with repeated measures, to study MLTC-M across the lifecourse. Future work may look to sequence LTCs and the sentinel condition on a larger scale and we hope this paper can be the foundation for this research.

Strengths and limitations:

The use of a prospective longitudinal approach provides the opportunity to analyse the time-sequenced nature of the data,[47]. Furthermore, there are multiple wider determinant exposures captured in BCS70, unavailable in most other datasets which will provide opportunities for future lifecourse investigation. Knowing the temporal sequencing of LTCs offers valuable means for elucidating an understanding of the accrual sequence of LTCs. However, the BCS70, like other major cohort studies, is affected by significant attrition and wave non-response. This is a limitation of our study – there were few people who reported both the most common three-condition MLTC-M at 46 and information on LTCs across all the adult sweeps (164/370). We therefore risk bias from underreporting of the MLTC-M group. Further, the small sample size also precludes the opportunity to explore men and women independently. However, this paper is exploratory: we do not conduct any regression analysis and are cautious not to overstate our findings. We recommend that future work using formal statistical methods consider accounting for attrition and wave non-response.

Data quality remains an important limitation. The variables used to assess the sequence of accrual of LTCs did not match up identically across sweeps and we were required to make assumptions about whether variables captured the same LTCs, and this may have accounted for some of the fluctuation in the reporting of psychological distress we observed. Further, at age 26, blood pressure was not recorded, although the number of those reporting high blood pressure by age 26 was likely to be small,[34,35]. Data coding constraints meant we were unable to provide a more precise measurement of when the sentinel condition was reported or differentiate between the times of onset of conditions if they were first recorded at the same sweep. Additionally, these data constraints meant our sequencing results were only granular down to the 4-year level. Further work in birth cohorts to harmonise and agree LTC definitions would strengthen their potential for multimorbidity research.

For peer review only - http://bmjopen.bmj.com/site/about/guidelines.xhtml

Some LTCs were self-reported and are therefore likely under-recorded, implying that we may be underestimating the true prevalence of multimorbidity. A further limitation of the data is that there may be inconsistency in the reporting of conditions across the sweeps of data collection. Additionally, the cohort is representative of a cohort of children born in 1970, and as such does not reflect the ethnically diverse population of the UK today (Supplementary Materials Table 1). It is known from previous research that the prevalence of multimorbidity is more common and occurs earlier in certain ethnic minority populations,[7,38]. We are also aware that health is not static and we aim to incorporate further methods capturing fluctuation in other LTCs when exploring the sequence of accrual of LTCs. Further, we focus on the relative risk of psychological distress; this measure is limited to symptoms of depression and anxiety, and does not capture the full range of other mental health problems. In addition, we define burdensomeness as psychological distress occurring as part of multimorbidity. However, there are other causes of burdensomeness such as physical symptoms and factors influencing treatment burden (number of health care appointments, polypharmacy and quality of life measures) we did not consider; these are described within the cumulative complexity model,[48] and burden treatment theory,[49]. These should be investigated in future work.

Finally, these analyses were exploratory and descriptive. Caution must be taken not to overstate our findings given the limitations of sample size, attrition and the fluctuation in the recording of LTCs. Limitations of these analyses precluded further statistical analysis. Future work should consider multiple wider determinant exposures captured in birth cohort datasets and explore the associations between the early lifecourse and both sentinel conditions and early onset burdensome multimorbidity clusters. However, more careful consideration around the role of confounders, mediators, attrition, sample size and the reporting of LTCs are required.

CONCLUSIONS

In these exploratory analyses in a national birth cohort, while overall prevalence of the most common three-condition burdensome multimorbidity group was relatively low (4.3%) at age 46, we identified psychological distress and back pain as the most common sentinel conditions. The MLTC-M group was characterised by more disadvantaged sociodemographic factors. Birth cohorts may usefully inform lifecourse-focused multimorbidity research but there are current limitations to their utility. We cannot influence the BCS70 sample size and diversity but future work could combine with other birth cohorts that include people into their 60s. Further work is also needed to agree on LTC definitions to strengthen their potential for multimorbidity research.

For peer review only - http://bmjopen.bmj.com/site/about/guidelines.xhtml

Figure Legend

Figure 1. Selection Flow of MLTC-M sample at age 46.

Figure 2. Bubble diagram of the percentage distribution of sentinel conditions and the age at which the sentinel condition was reported, for all 164 cohort members who reported the MLTC-M group of psychological distress, high blood pressure and back pain at age 46 and provided information on LTC across the adult sweeps. *The area of each bubble represents, for those that reported the MLTC-M group of psychological distress, high blood pressure and back pain at age 46, the reporting of the sentinel condition at each sweep in adulthood. The percentages are included inside each bubble.*

Figure 3. Sankey diagram of sequences of long-term condition for all 164 cohort members who reported the MLTC-M group of psychological distress (PD), high blood pressure (BP) and back pain (pain) at age 46 and provided information on LTC across the adult sweeps. *The size of the bar represents the number of people within each pathway. Larger bars representing a greater number of people.*

Figure 4. A graph representing the reporting of psychological distress across the adult sweeps for all 164 cohort members who reported the MLTC-M group of psychological distress, high blood pressure and back pain at age 46 and provided information on LTC across the adult sweeps.

For peer review only

Statements

Contributorship: SS, EH, NA, MB, RH, ZZ, SC and SF were involved in the conceptualisation of ideas. SF obtained the BCS70 data. SS, EH, NA, and SF designed and planned the analyses. SS and EH performed the statistical analysis. SS initial draft of manuscript. SS, EH, NA, SC, RH, MB, WW, JM, MA and SF were involved in drafting, editing and reviewing the manuscript. All authors contributed to the development of the project and were involved in the approval of the final manuscript. SS and SF take responsibility for the data and research governance.

Funding statement: This research is supported by NIHR grant number [NIHR202644] as part of a larger research project titled *'Developing a Multidisciplinary Ecosystem to Study Lifecourse Determinants of Complex Mid-life Multimorbidity using Artificial Intelligence (MELD)'*. The funder of the study had no role in the study design, data collection, data analysis, data interpretation, or the writing of the paper. The views and opinions expressed in this paper are those of the authors and do not necessarily reflect those of the NIHR or the Department of Health and Social Care.

Competing interests: All authors (SS, EH, NA, MB, RH, ZZ, SC, MA, SF, JM and WW) have no competing interests.

Ethical approval: Ethical approval for the BCS70 study was granted by the National Health Service (NHS) Research Ethics Committee, and all participants provided fully informed consent. For details on the BCS70 ethics and consent please see: https://cls.ucl.ac.uk/wp-content/uploads/2017/07/BCS70-ethical-review-and-consent-Shepherd-P-November-2012.pdf. Ethics approval for the MELD project which this analysis forms part of was granted by Southampton University Ethics and Research Governance Online (61451) and by The Proportionate Review Sub-committee of the London - Camden & Kings Cross Research Ethics Committee (21/PR/0023).

Data sharing: The BCS70 datasets generated and analysed in the current study are available in the UK Data Service repository: BCS70 https://discover.ukdataservice.ac.uk/series/?sn=200001.

For peer review only

For peer review only - http://bmjopen.bmj.com/site/about/guidelines.xhtml

Reference List:

1. Kingston A, Robinson L, Booth H, et al. Projections of multi-morbidity in the older population in England to 2035: estimates from the population ageing and care simulation (PACSim) model. *Age Ageing* 2018;47(3):374–80.

2. Whitty CJM, MacEwen C, Goddard A, et al. Rising to the challenge of multimorbidity. *BMJ* 2020; 368:l6964.

3. Barnett K, Mercer S W, Norbury M, et al. Epidemiology of multimorbidity and implications for health care, research, and medical education: a cross-sectional study. *Lancet* 2012; 380(9836):37-43.

4. The Academy of Medical Sciences. Multimorbidity: a priority for global health research. London: Academy of Medical Sciences; 2018 [accessed 2021 01/09]. Available from: https://acmedsci.ac.uk/policy/policy-projects/multimorbidity

5. Moussavi S, Chatterji S, Verdes E, et al. Depression, chronic diseases, and decrements in health: Results from the World Health Surveys. *The Lancet* 2007;370:851–858.

6. Pescosolido BA, Patient trajectories. In Cockerham WC, Dingwall R, Quah SR eds. The Wiley Blackwell Encyclopedia of Health, Illness, Behavior, and Society, First Edition. John Wiley & Sons, Ltd 2014:1-8.

7. Ashworth M, Durbaba S, Whitney D, et al. Journey to multimorbidity: longitudinal analysis exploring cardiovascular risk factors and sociodemographic determinants in an urban setting. *BMJ Open* 2019;9(12):e031649.

8. Fortin M, Stewart M, Poitras ME, et al. A systematic review of prevalence studies on multimorbidity: toward a more uniform methodology. *Ann Fam Med* 2012;10(2):142–51.

9. Mujica-Mota RE, Roberts M, Abel G, et al. Patterns of morbidity and multi-morbidity and their impact on health-related quality of life: evidence from a national survey. *Qual Life Res* 2015;24(4):909–18.

10. Rhebergen D, Batelaan NM, de Graaf R, et al. The 7-year course of depression and anxiety in the general population. *Acta Psychiatrica Scandinavica* 2011; 123: 297-306.

11. Gustavson K, Knudsen AK, Nesvåg R, et al. Prevalence and stability of mental disorders among young adults: Findings from a longitudinal study. *BMC Psychiatry* 2018; 18, 65.

12. Lamers F, Beekman AT, van Hemert AM, et al. Six-year longitudinal course and outcomes of subtypes of depression. *Br J Psychiatry* 2016;208(1):62–8.

13. Brodaty H, Luscombe G, Peisah C, et al. A 25-year longitudinal, comparison study of the outcome of depression. *Psychol Med* 2001;31:1347–1359.

For peer review only - http://bmjopen.bmj.com/site/about/guidelines.xhtml

14. Mueller TI, Leon AC, Keller MB, et al. Recurrence after recovery from major depressive disorder during 15 years of observational follow-up. *Am J Psychiatry* 1999;156:1000–1006.

15. Roshanaei-Moghaddam B, Katon WJ, & Russo J. The longitudinal effects of depression on physical activity. *Ann Gen Hosp Psychiatry* 2009; 31(4), 306–315.

16. Doering LV, Moser DK, Riegel B, et al. Persistent comorbid symptoms of depression and anxiety predict mortality in heart disease. *Int J Cardiol* 2010;145(2):188-192.

17. Mercer SW, Gunn J, Bower P, et al. Managing patients with mental and physical multimorbidity. *BMJ* 2012;345:e5559.

18. Multimorbidity: clinical assessment and management NICE guideline [NG56] Published: 21 September 2016 [accessed 2021 01/09]. Available from https://www.nice.org.uk/guidance/ng56.

19. Gondek D, Bann D, Brown M, et al. Prevalence and early-life determinants of mid-life multimorbidity: evidence from the 1970 British birth cohort. *BMC Public Health* 2021; 1319.

20. UK Data Service. 1970 British Cohort Study [accessed 2022 30/08]. Available from: beta.ukdataservice.ac.uk/datacatalogue/series/series?id=200001

21. Elliott J, & Shepherd P. Cohort profile: 1970 British Birth Cohort (BCS70). *Int J Epidemiol* 2006;35(4):836–43.

22. (UCL) Centre For Longitudinal Studies. User guides 2019 [Accessed 2019 01/05]. Available from: https://cls.ucl.ac.uk/data_documentation/?types%5B%5D=user-guides&studies%5B%5D&studies%5B%5D=bcs70-birth-sweep&studies%5B%5D=age-5-sweep&studies%5B%5D=age-10-sweep&studies%5B%5D=age-16-sweep-1970-british-cohort-study&studies%5B%5D=age-26-sweep&studies%5B%5D=age-30-sweep&studies%5B%5D=age-34-sweep&studies%5B%5D=age-38-sweep&studies%5B%5D=age-42-sweep-1970-british-cohort-study&studies%5B%5D=age-46-biomedical-sweep&s=&filter=1

23. UCL – 1970 British Cohort Study [accessed 2021 01/09]. Available from: https://cls.ucl.ac.uk/cls-studies/1970-british-cohort-study/

24. Holland E, Stannard S, Alwan N, et al. Exploring sentinel conditions and the accrual sequence of multiple long-term condition multimorbidity using birth cohort and primary care data: an exploratory retrospective cohort study [abstract]. *Lancet* 2021; 398:S54.

25. UK Data Service [accessed 2021 05/09]. Available from :https://ukdataservice.ac.uk//app/uploads/teachingresource1970bcsmalaiseinventory.pdf

For peer review only

26. Gondek D, Bann D, Patalay P, et al. Psychological distress from early adulthood to early old age: Evidence from the 1946, 1958 and 1970 British birth cohorts. *Psychol Med* 2021; 1-10.

27. Mirowsky J, & Ross CE. Measurement for a human science. *J Health Soc Behav* 2002; 43, 152–170.

28. Ploubidis GB, Sullivan A, Brown M, et al. Psychological distress in midlife: Evidence from the 1958 and 1970 British birth cohorts. *Psychol Med* 2017; 47, 291–303.

29. Ploubidis GB, McElroy E, & Moreira H C. A longitudinal examination of the measurement equivalence of mental health assessments in two British birth cohorts. *Longit Life Course Stud* 2019; 10, 471–489.

30. McGee R, Williams S, & Silva PA. An evaluation of the malaise inventory. *J Psychosom Res*1986; 30, 147–152.

31. Furnham A, & Cheng H. The stability and change of malaise scores over 27 years: Findings from a nationally representative sample. *Pers Individ Dif* 2015; 79, 30–34.

32. Rodgers B, Pickles A, Power C, et al. Validity of the Malaise Inventory in general population samples. *Soc Psychiatry and Psychiatr Epidemiol* 1999; 34, 333–341.

33. Messerli F, Rimoldi S, & Bangalore S. Changing definition of hypertension in guidelines: how innocent a number game? *Eu Heart J* 2018; 39(24): 2241–2242.

34. Lewington S, Clarke R, Qizilbash N, et al. Age-specific relevance of usual blood pressure to vascular mortality: a meta-analysis of individual data for one million adults in 61 prospective studies. *Lancet* 2002; 360(9349):1903–13.

35. NHS Digital. Health Survey for England: High blood pressure [accessed 2021 10/11]. Available from: http://healthsurvey.hscic.gov.uk/data-visualisation/data-visualisation/explore-the-trends/high-blood-pressure.aspx

36. Jsfiddle [accessed 2021 01/09]. Available from: https://jsfiddle.net

37. StataCorp. 2019. *Stata Statistical Software: Release 16*. College Station, TX: StataCorp LLC.

38. St Sauver JL, Boyd CM, Grossardt BR, et al. Risk of developing multimorbidity across all ages in an historical cohort study: differences by sex and ethnicity. *BMJ Open;* 2015;5.

39. Cassell A, Edwards D, Harshfield A, et al. The epidemiology of multimorbidity in primary care: a retrospective cohort study. *Br J Gen Pract* 2018; 68 (669): e245-e251.

40. Barnett K, Mercer S W, Norbury M, et al. Epidemiology of multimorbidity and implications for health care, research, and medical education: a cross-sectional study. *Lancet* 2012; 380(9836):37-43.

For peer review only - http://bmjopen.bmj.com/site/about/guidelines.xhtml

41. Xu X, Mishra GD, Dobson AJ, et al. Progression of diabetes, heart disease, and stroke multimorbidity in middle-aged women: A 20-year cohort study. *PLoS Med* 2018; 15(3): e1002516.

42. Ruel G, Lévesque JF, Stocks N, et al. Understanding the Evolution of Multimorbidity: Evidences from the North West Adelaide Health Longitudinal Study (NWAHS). *PLoS One* 2014; 9(5): e9629.

43. Holgate ST, & Davies DE. Rethinking the Pathogenesis of Asthma, *Immunity* 2009; 31(3), 362-367.

44. Dunn KM, Jordan K, & Croft PR, Characterising the course of low back pain: a latent class analysis. *Am J Epidemiol*. 2006; 163: 754-761.

45. Carmen D, & Cunningham SA. In, out, and fluctuating: obesity from adolescence to adulthood, *Ann Epidemiol* 2020; 41: 14-20.

46. Morris JE, Roderick PJ, Harris S, et al. Treatment burden for patients with multimorbidity: cross-sectional study with exploration of a single-item measure. *Br J Gen Pract* 2021; 71 (706): e381-e390.

47. Wadsworth, M. & Bynner, J. A Companion to Life Course Studies The social and historical context of the British birth cohort studies Introduction. *Routledge Advanced Sociology* 2011; 1-18.

48. Shippee ND, Shah ND, May CR, et al. Cumulative complexity: a functional, patient-centered model of patient complexity can improve research and practice. *J Clin Epidemiol* 2012; 65(10), 1041–1051.

49. May CR, Eton DT, Boehmer K, et al. Rethinking the patient: using Burden of Treatment Theory to understand the changing dynamics of illness. *BMC Health Serv Res* 2014; 14, 281.

For peer review only

Figure 1. Selection Flow of MLTC-M sample at age 46

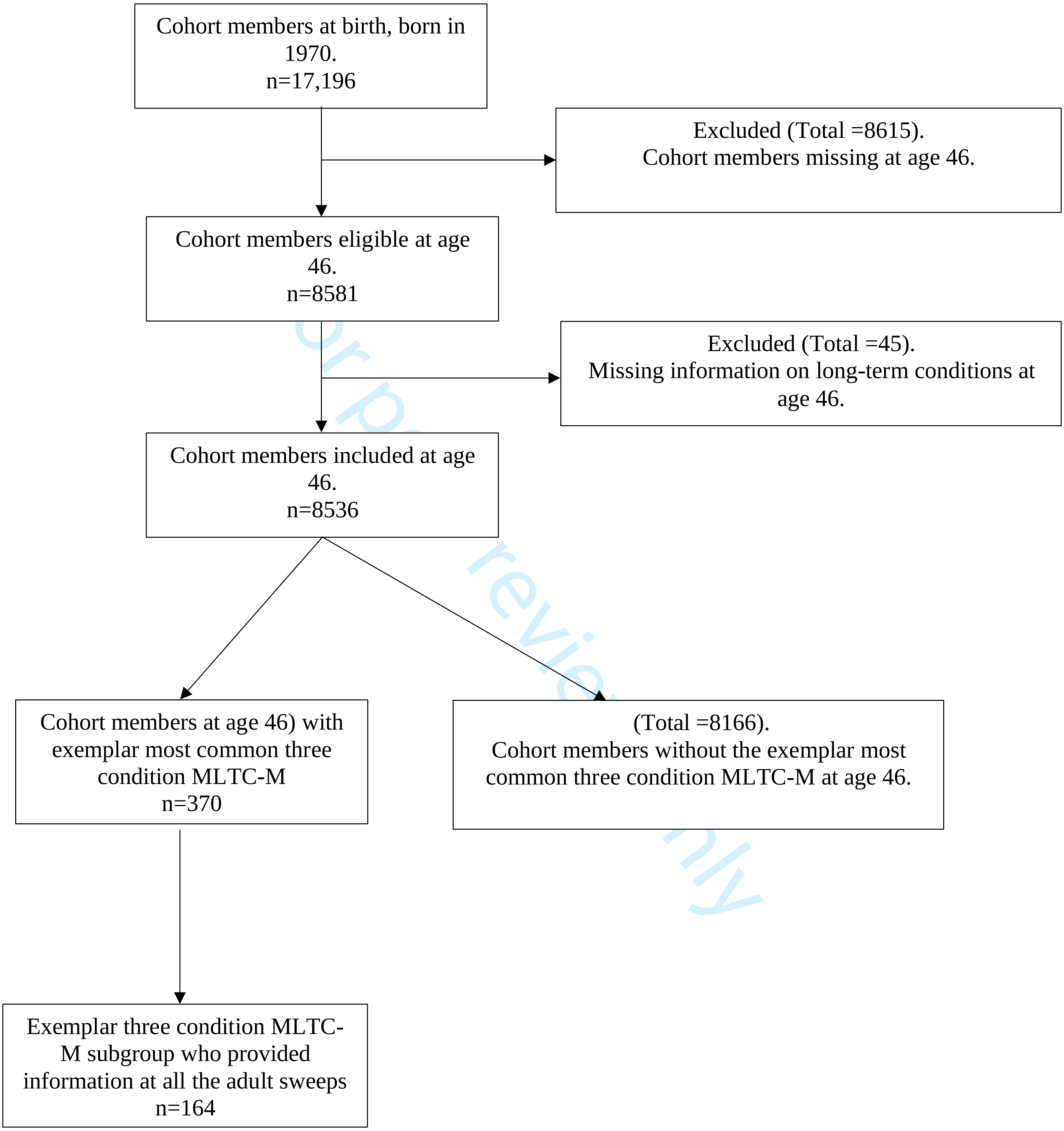

Figure 2. Bubble diagram of the percentage distribution of sentinel conditions and the age at which the sentinel condition was reported, for all 164 cohort members who reported the MLTC-M group of psychological distress, high blood pressure and back pain at age 46 and provided information on LTC across the adult sweeps.

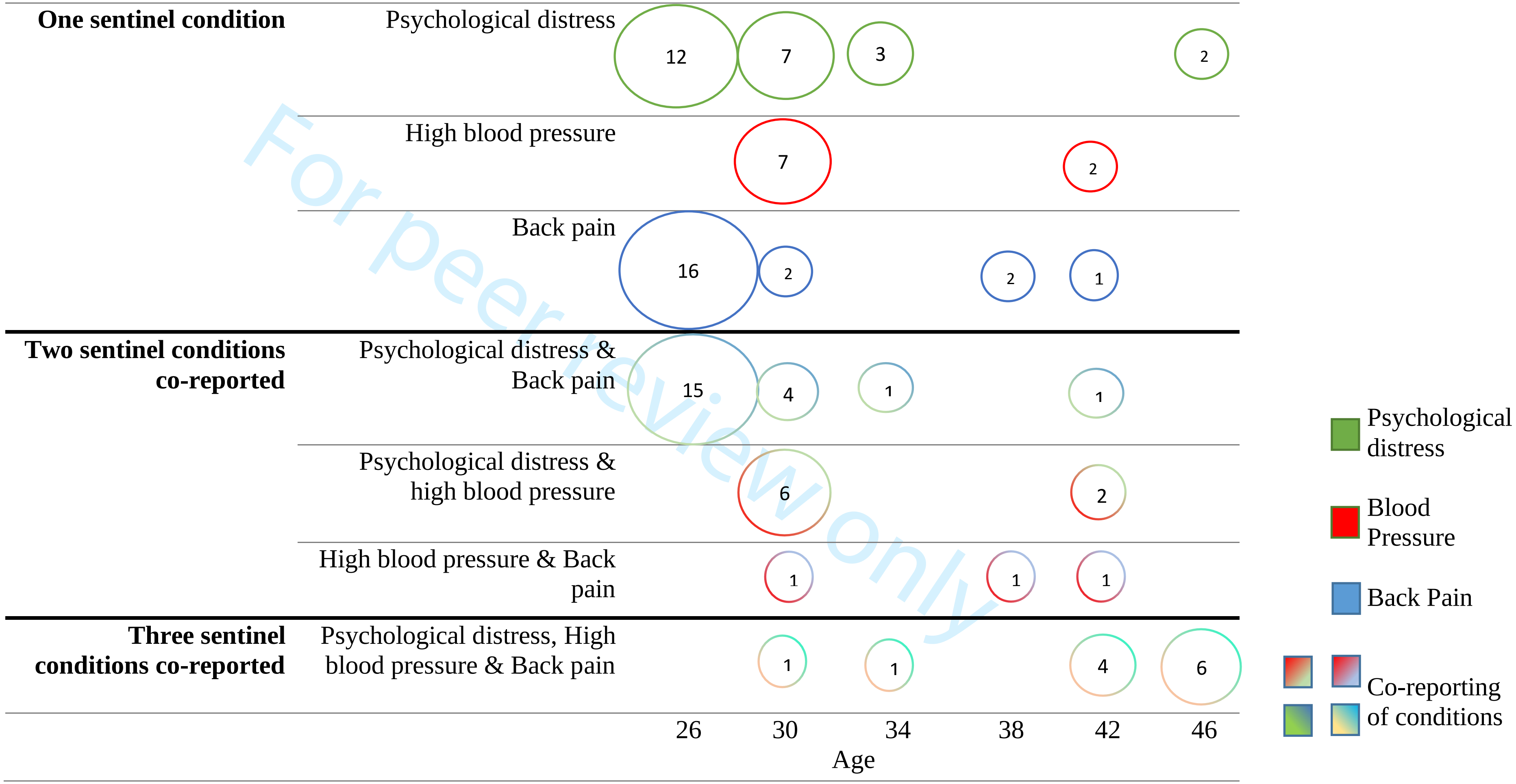

The area of each bubble represents, for those that reported the MLTC-M group of psychological distress, *high blood pressure and back pain at age 46, the reporting of the sentinel condition at each sweep in adulthood. The percentages are included inside each bubble.*

Figure 3. Sankey diagram of sequences of long-term condition for all 164 cohort members who reported the MLTC-M group of psychological distress (PD), high blood pressure (BP) and back pain (pain) at age 46 and provided information on LTC across the adult sweeps.

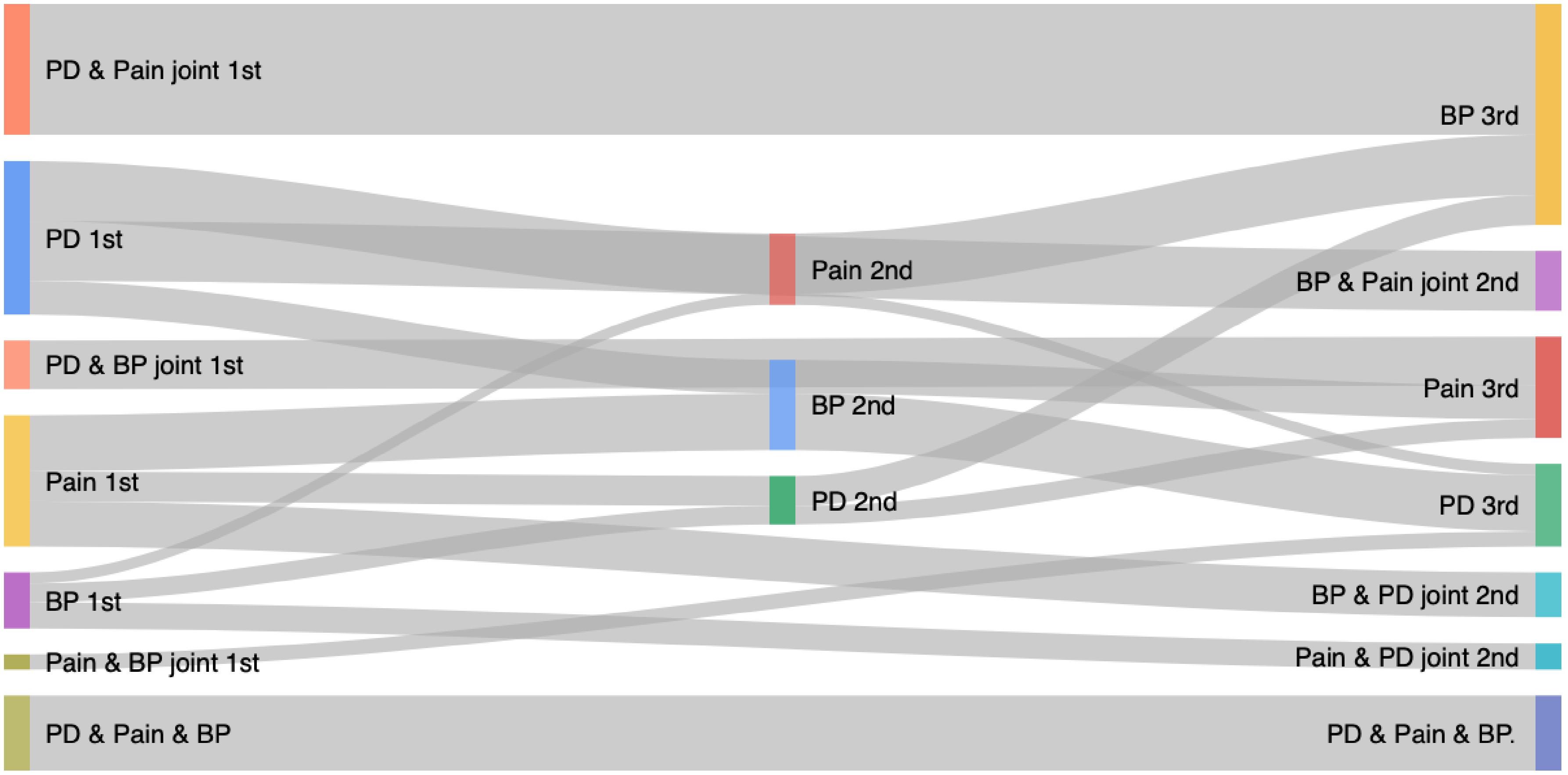

The size of the bar represents the number of people within each pathway. Larger bars representing a greater number of people.

Figure 4. A graph representing the reporting of psychological distress across the adult sweeps for all 164 cohort members who reported the MLTC-M group of psychological distress, high blood pressure and back pain at age 46 and provided information on LTC across the adult sweeps.

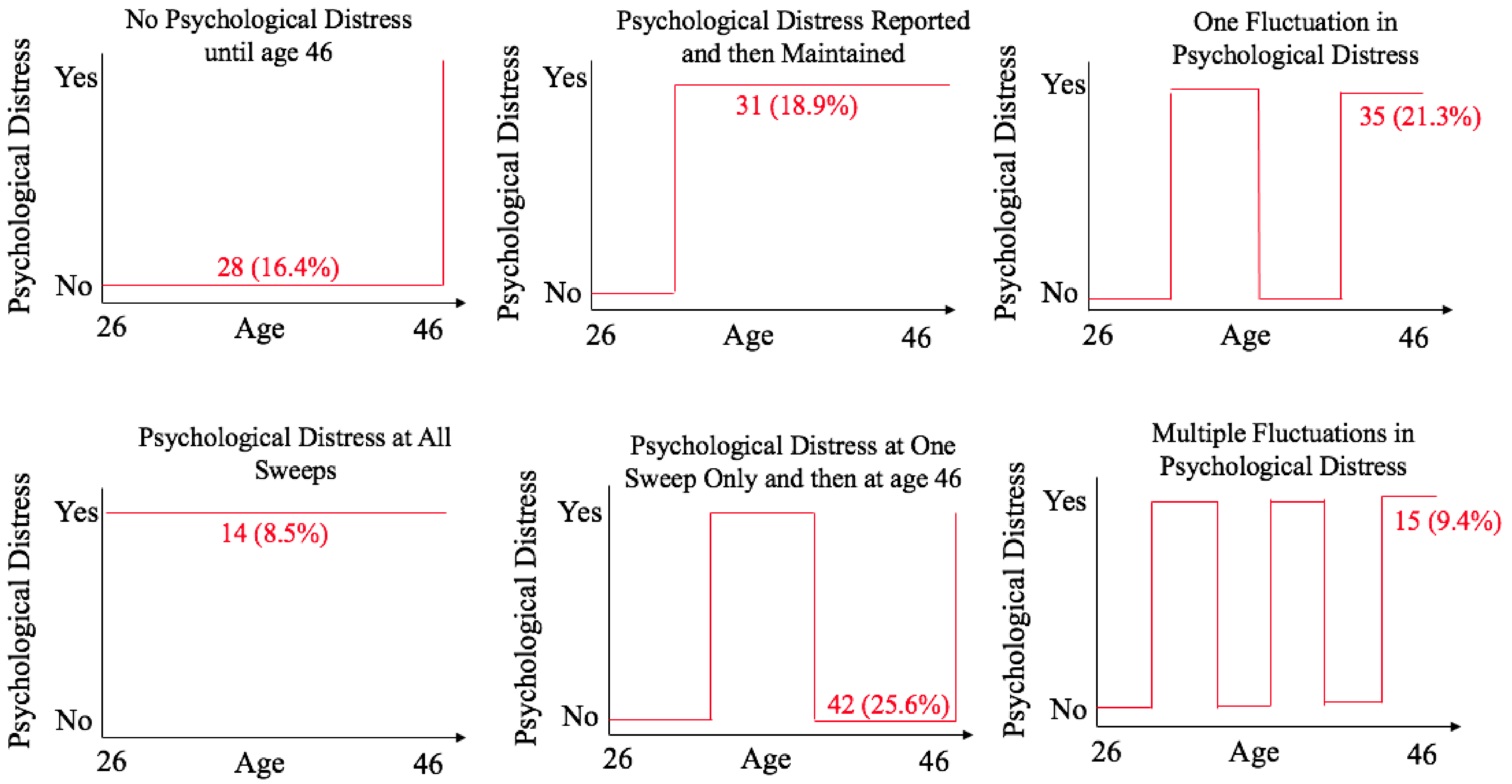

Supplementary Materials

Table 1. Ethnicity of the cohort members at age five[1] for MLTC-M group (psychological distress, high blood pressure and back pain), compared with remainder of BCS70 sample at age 46.

		MLTC-M	Non MLTC-M
		N (%)	N (%)
Ethnicity	White British	276 (94.5%)	6268 (96.7%)
	European	5 (1.7%)	58 (0.9%)
	West Indian	4 (1.4%)	52 (0.8%)
	Indian-Pakistani	5 (1.7%)	77 (1.2%)
	Other Asian	0 (0%)	8 (0.1%)
	African	1 (0.3%)	1 (0.0%)
	Other	1 (0.3%)	16 (0.25%)
	Total	292 (100%)	6480 (100%)

[1]*Ethnicity of the cohort member was not recorded at birth, age 5 was the first sweep where ethnicity was reported.*

For peer review only

Reporting checklist for cohort study.

Based on the STROBE cohort guidelines.

Instructions to authors

Complete this checklist by entering the page numbers from your manuscript where readers will find each of the items listed below.

Your article may not currently address all the items on the checklist. Please modify your text to include the missing information. If you are certain that an item does not apply, please write "n/a" and provide a short explanation.

Upload your completed checklist as an extra file when you submit to a journal.

In your methods section, say that you used the STROBE cohortreporting guidelines, and cite them as:

von Elm E, Altman DG, Egger M, Pocock SJ, Gotzsche PC, Vandenbroucke JP. The Strengthening the Reporting of Observational Studies in Epidemiology (STROBE) Statement: guidelines for reporting observational studies.

		Reporting Item	Page Number
**Title and abstract**			
Title	#1a	Indicate the study's design with a commonly used term in the title or the abstract	1
Abstract	#1b	Provide in the abstract an informative and balanced summary of what was done and what was found	2
**Introduction**			
Background / rationale	#2	Explain the scientific background and rationale for the investigation being reported	3
Objectives	#3	State specific objectives, including any prespecified hypotheses	4
**Methods**			
Study design	#4	Present key elements of study design early in the paper	5

For peer review only

Setting	#5	Describe the setting, locations, and relevant dates, including periods of recruitment, exposure, follow-up, and data collection	5
Eligibility criteria	#6a	Give the eligibility criteria, and the sources and methods of selection of participants. Describe methods of follow-up.	5
Eligibility criteria	#6b	For matched studies, give matching criteria and number of exposed and unexposed	n/a
Variables	#7	Clearly define all outcomes, exposures, predictors, potential confounders, and effect modifiers. Give diagnostic criteria, if applicable	6
Data sources / measurement	#8	For each variable of interest give sources of data and details of methods of assessment (measurement). Describe comparability of assessment methods if there is more than one group. Give information separately for for exposed and unexposed groups if applicable.	5
Bias	#9	Describe any efforts to address potential sources of bias	5
Study size	#10	Explain how the study size was arrived at	5
Quantitative variables	#11	Explain how quantitative variables were handled in the analyses. If applicable, describe which groupings were chosen, and why	5
Statistical methods	#12a	Describe all statistical methods, including those used to control for confounding	
5			
Statistical methods	#12b	Describe any methods used to examine subgroups and interactions	7
Statistical methods	#12c	Explain how missing data were addressed	7
Statistical methods	#12d	If applicable, explain how loss to follow-up was addressed	n/a
Statistical methods	#12e	Describe any sensitivity analyses	
13			

Results

For peer review only

Participants	#13a	Report numbers of individuals at each stage of study—eg numbers potentially eligible, examined for eligibility, confirmed eligible, included in the study, completing follow-up, and analysed. Give information separately for for exposed and unexposed groups if applicable.	7
Participants	#13b	Give reasons for non-participation at each stage	7
Participants	#13c	Consider use of a flow diagram	
7			
Descriptive data	#14a	Give characteristics of study participants (eg demographic, clinical, social) and information on exposures and potential confounders. Give information separately for exposed and unexposed groups if applicable.	7
Descriptive data	#14b	Indicate number of participants with missing data for each variable of interest	
7			
Descriptive data	#14c	Summarise follow-up time (eg, average and total amount)	
7			
Outcome data	#15	Report numbers of outcome events or summary measures over time. Give information separately for exposed and unexposed groups if applicable.	
8			
Main results	#16a	Give unadjusted estimates and, if applicable, confounder-adjusted estimates and their precision (eg, 95% confidence interval). Make clear which confounders were adjusted for and why they were included	9
Main results	#16b	Report category boundaries when continuous variables were categorized	9
Main results	#16c	If relevant, consider translating estimates of relative risk into absolute risk for a meaningful time period	
n/a			

For peer review only

Other analyses	#17	Report other analyses done—eg analyses of subgroups and interactions, and sensitivity analyses	9
**Discussion**			
Key results	#18	Summarise key results with reference to study objectives	12
Limitations	#19	Discuss limitations of the study, taking into account sources of potential bias or imprecision. Discuss both direction and magnitude of any potential bias.	13
Interpretation	#20	Give a cautious overall interpretation considering objectives, limitations, multiplicity of analyses, results from similar studies, and other relevant evidence.	12
Generalisability	#21	Discuss the generalisability (external validity) of the study results	13
**Other Information**			
Funding	#22	Give the source of funding and the role of the funders for the present study and, if applicable, for the original study on which the present article is based	14

The STROBE checklist is distributed under the terms of the Creative Commons Attribution License CC-BY. This checklist was completed on 23. November 2021 using https://www.goodreports.org/, a tool made by the EQUATOR Network in collaboration with Penelope.ai

For peer review only
